# Improving the Performance of Photoactive Terpene-Based Resin Formulations for Light-Based Additive Manufacturing

**DOI:** 10.1021/acssuschemeng.3c08191

**Published:** 2024-04-24

**Authors:** Viviane Chiaradia, Elena Pensa, Thiago O. Machado, Andrew P. Dove

**Affiliations:** School of Chemistry, University of Birmingham, Edgbaston, Birmingham B15 2TT, United Kingdom

**Keywords:** terpenes, itaconic anhydride, thiol−ene reactions, renewable resins, reactive diluents, 3D printing, digital light processing

## Abstract

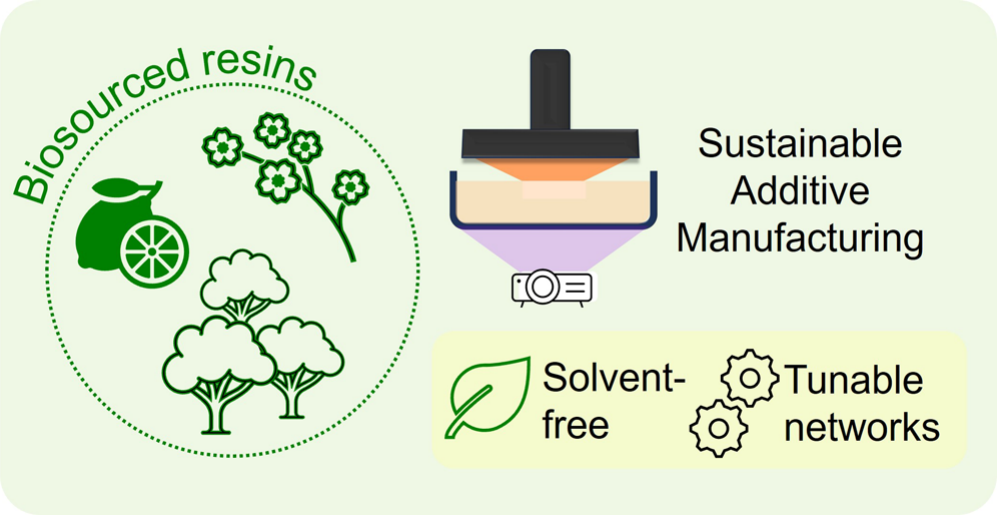

Photocurable liquid formulations have been a key research focus for the preparation of mechanically robust and thermally stable networks. However, the development of renewable resins to replace petroleum-based commodities presents a great challenge in the field. From this perspective, we disclose the design of photoactive resins based on terpenes and itaconic acid, both potentially naturally sourced, to prepare photosets with adjustable thermomechanical properties. Biobased perillyl itaconate (PerIt) was synthesized from renewable perillyl alcohol and itaconic anhydride via a scalable solvent-free method. Photoirradiation of PerIt in the presence of a multiarm thiol and photoinitiator led to the formation of networks over a range of compositions. Addition of nonmodified terpenes (perillyl alcohol, linalool, or limonene) as reactive diluents allowed for more facile preparation of photocured networks. Photosets within a wide range of properties were accessed, and these could be adjusted by varying diluent type and thiol stoichiometry. The resins showed rapid photocuring kinetics and the ability to form either brittle or elastic materials, with Young’s modulus and strain at break ranging from 3.6 to 358 MPa and 15 to 367%, respectively, depending on the chemical composition of the resin. Glass transition temperatures (*T*_g_) were influenced by thioether content, with temperatures ranging from 5 to 43 °C, and all photosets displayed good thermal resistance with *T*_d,5%_ > 190 °C. Selected formulations containing PerIt and limonene demonstrated suitability for additive manufacturing technologies and high-resolution objects were printed via digital light processing (DLP). Overall, this work presents a simple and straightforward route to prepare renewable resins for rapid prototyping applications.

## Abstract 2

Resin formulations based on biobased terpenes were investigated to offer a simple, scalable, and environmentally friendly route for rapid photocuring.

## Introduction

Covalently cross-linked materials are highly desirable as a consequence of their excellent thermal stability and tunable mechanical strength that make them suitable for applications ranging from adhesives to robust biomedical devices. The ability to form these materials by exposing photoactive resins to light has motivated their study in application areas as diverse as coatings and additive manufacturing.^1^ Despite their importance, the range of photoactive monomers that have been explored are limited and most commercial resins are based on petrochemical feedstocks.^2,3^ In turn, the resultant materials are largely not degradable. Growing environmental awareness has led to a desire to move away from petrochemically sourced chemicals and has boosted research toward renewable monomers to develop sustainable alternatives and reduce both the carbon footprint and reliance on fossil-fuel sources.^4^

To address the increased demand for sustainable feedstocks, monomers from vegetable oils,^5−7^ terpenes,^8−10^ lignin derivatives,^11,12^ lactones, and lactides,^13−15^ as well as bioderived (meth)acrylates,^7,16,17^ have emerged as alternatives to generate materials with competitive or superior properties compared to those of nonrenewable commodities.^4,18,19^ However, the design of new polymeric networks with high biobased content and nonlaborious synthesis while simultaneously applying green chemistry principles is a critical challenge in the field. Hence, the translation of these systems to industrial use is still beset with these limitations and more advanced strategies are required to prepare petrochemical-free plastics.^4^

On account of their functionalities and ease of modification, vegetable oils that are modified with acrylates, epoxides, or methacrylates have been widely investigated for photoactive resin formulations.^20,21^ The desirable features of (meth)acrylates, which include low cost, wide availability, and rapid photocuring, have made them key for designing cross-linked materials. In fact, there is a vast number of studies combining (meth)acrylates with biomass-derived monomers to deliver more sustainable systems.^7,12,22^ For example, Zheng and co-workers have reported the design of di- and trifunctional acrylates, where glycidyl methacrylate was reacted with either succinic acid or itaconic acid to form reactive liquid monomers in a single-step and solvent-free reaction.^22^ Terpenes and terpenoids are another class of biobased chemicals that have gained increased prominence on account of their versatile reactivity. They can be easily accessed in large scale from essential oils or citrus fruits or as byproducts of different industries.^4^ Several works have reported their modification and polymerization, as well as postpolymerization strategies to design either elastomers or polymeric networks with tailored properties.^19^ Some examples include cationic polymerization,^23,24^ modification with acrylates,^11,25^ ring-opening polymerization,^26,27^ and thiol–ene additions.^9,28−30^ This latter method is well described and has been extensively used for UV-initiated photopolymer systems, as thiols are widely available commercially and form homogeneous photosets via a step-growth mechanism under photoirradiation. Previously in our group, we described the thiol-initiated photopolymerization of a series of terpenes and terpenoids, including limonene, linalool, nerol, geraniol, and myrcene. Formulation of each of the terpene monomers with a tetrafunctional thiol led to the observation of different reactivities that were related to the accessibility of the double bonds within the terpene. While 93% of the *exo*-alkene moieties of limonene were converted upon photoirradiation, only 27% conversion was observed for the *endo*-alkene moieties. As a consequence of the reactivity differences, most of the networks displayed limited mechanical performance and a mixture of oligomeric and nonmodified terpenes was required for applications such as 3D printing.^29,30^

With the desire to expand the toolbox of available photoactive monomers while overcoming the synthetic limitations of many current approaches to incorporate terpene and terpenoid monomers into photocurable resins, we sought to investigate the modification of a terpenoid containing a hydroxyl functionality that allows for easy modification via traditional esterification routes. With these principles in mind, we describe a simple yet effective approach to generating renewable resin formulations based on a modified terpenoid alcohol. The biobased resins were accessed via solvent-free methods to afford photosets with adjustable thermomechanical properties. Properties such as photocuring kinetics, *T*_*g*_, and tensile were adjusted by varying the stoichiometry of thiol in the resin formulations, as well as the reactive diluent type. To further expand the scope of the work, resins displaying different photoreactivity and viscosities were employed to create preliminary 3D objects, and selected formulations found promising utility in DLP application.

## Experimental Section

### Materials

All compounds, unless otherwise indicated, were purchased from commercial sources and used as received.

### NMR Spectroscopy analysis

All NMR spectroscopy experiments were performed at 300 K on a Bruker DPX-400 NMR instrument equipped with an operating frequency of 400 MHz for ^1^H (100.57 MHz for ^13^C). ^1^H NMR spectra are referenced to residual protic solvent (δ = 7.26 for CDCl_3_) and ^13^C NMR spectra are referenced to the solvent signal (δ = 77.16 for CDCl_3_). The resonance multiplicities are described as s (singlet), d (doublet), t (triplet), q (quartet), or m (multiplet).

### Mass Spectrometry

High resolution electrospray ionization mass spectrometry was performed in the School of Chemistry at the University of Birmingham on a Waters Xevo G2-XS QTof Quadrupole Time-of-Flight mass spectrometer.

### Photorheology

The cross-linking kinetics of the resins were examined as a function of gelation time by photorheology using an Anton Paar MCR-302 rheometer fitted with a detachable photoillumination system (Exfo OmniCure S1500 UV light source, broadband HG-lamp, glass plate). Resin samples were sheared between two 25 mm parallel plates (0.25 mm gap width) at 0.5–2 Hz with an amplitude of 1% for 50 s without irradiation. After this time, the light source was switched on manually, and measurements were recorded every 0.2 s over ≤10 min. The intersection of the storage and loss moduli plots was taken as the gelation point of the resin.

### Rheology

Viscosity measurements were performed on an Anton Paar MCR-302 rheometer equipped with PP50 parallel plate geometry at room temperature. Resin formulations were loaded directly onto the plate, and the gap width was set at 1 mm. Shear rate sweeps were performed from 0.1 to 100 s^–1^ for 5 s per shear rate to determine the viscosity.

### Differential Scanning Calorimetry (DSC)

Thermal characterization of the networks was determined using differential scanning calorimetry (STARe system DSC3, Mettler Toledo) from −20 to 150 °C at a heating rate of 10 °C·min^–1^ for two heating/cooling cycles unless otherwise specified. The glass transition temperature (*T*_g_) was determined by the minimum of the first derivative and the melting point (*T*_m_*)* from the endothermic peak value in the second heating cycle of the DSC.

### Thermogravimetric Analysis (TGA)

TGA thermograms were obtained using a Q50 thermogravimetric analyzer (Mettler Toledo). Thermograms were recorded under an N_2_ atmosphere at a heating rate of 10 °C·min^–1^, from 25 to 600 °C, with an average sample weight of *ca*. 5 mg. Aluminum pans were used for all samples. Decomposition temperatures were reported as the 5% weight loss temperature (*T*_d,5%_).

### Dynamic Mechanical Analysis (DMA)

Dynamic mechanical thermal analysis (DMTA) data were obtained using a Mettler Toledo DMA 1 star system and analyzed using the software package STARe V13.00a. Thermal sweeps were conducted using films (W × thickness = 1.6 mm × 0.7 mm) cooled to −120 °C and held isothermally for ca. 5 min. Storage and loss moduli, as well as the loss factor (ratio of E″ and E′, tan δ) were probed as the temperature was swept from −120 to 150 °C, 5 °C·min^–1^.

### Fourier-Transform Infrared (FTIR) Spectroscopy

FTIR spectra were collected using an Agilent Technologies Cary 630 FTIR spectrometer. Sixteen scans from 600 to 4000 cm^–1^ were taken at a resolution of 2 cm^–1^, and the spectra were corrected for background absorbance.

### Uniaxial Tensile Strength Testing

All uniaxial tensile tests were performed on a Testometric M350–-5CT universal mechanical testing instrument fitted with a load cell of 5 kg F. Measurements were performed at ambient temperature on thin films (ca. 0.7 mm) cut into dog-bone-shaped samples using an ASTM Type IV Die with a die cutter. The sample width (ca. 1.6 mm) and thickness (ca. 0.7 mm) were measured for each individual sample, and the average value was recorded before mechanical analyses were conducted. Each dog-bone specimen was clamped into the tensile grips and subjected to an elongation rate of 10 mm min^–1^ until failure. For each sample formulation, at least 5 dog-bones were tested with the mean average being reported.

### Gel Fraction Testing

Postcured films were cut into squares (ca. 5 × 5 × 0.7 mm) and weighed (*W*_i_) before being submerged in 2 mL of THF and allowed to swell until equilibrium was reached after 24 h. The swollen films were then dried and weighed (*W*_d_), and eq 1 was used to determine the gel fraction (%). Experiments were repeated in triplicate, and mean values were reported.
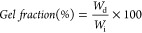
1

### Digital Light Processing (DLP) 3D Printing

The printing of high-resolution structures was carried out by using a MiiCraft Ultra 50 3D printer with a 405 nm LED light source. To access the Z-cure depth of each resin, formulations were poured onto glass slides, and a square shape was irradiated for 40–110 s. Excess of noncured resin was removed using a tissue, and the cure depth of each square was measured using a micrometer. After accessing depth cure, resins were used to print objects in accordance with a 3D CAD model (squares and rectangles with features) with a slice thickness of 25 or 50 μm at different layer curing times. The features of each print were visualized using an Alicona G4 InfiniteFocus system, and the images were processed using ImageJ 1.53a.

### Synthesis of Perillyl Itaconate

Itaconic anhydride (8.46 g, 75.48 mmol) and perillyl alcohol (11.49 g, 75.48 mmol) were placed in a 50 mL single-neck round-bottom flask in a 1:1 molar ratio. The mixture was heated up to 50 °C and the catalyst (Novozym 435, 2.5 wt % of total reagents) was added. The reaction was continuously monitored using ^1^H NMR and TLC (50:50 EtOAc:Hexane) and at the appropriate conversion the mixture was filtered to remove the enzyme and dried. After purification via silica column chromatography using DCM/MeOH as an eluent (90:10), perillyl itaconate was obtained as a light-yellow solid (15.2 g, 86% yield).

^1^H NMR (400 MHz, CDCl_3_) δ 6.40 (dd, *J* = 34.4, 0.9 Hz, 1H), 5.86–5.78 (m, 1H), 5.78–5.69 (m, 1H), 4.75–4.64 (m, 2H), 4.52 (d, J = 28.0 Hz, 2H), 3.42–3.28 (m, 2H), 2.21–1.40 (m, 10H). ^13^C NMR (101 MHz, CDCl_3_) δ: 171.91, 170.91, 150.01, 133.75, 132.79, 131.17, 126.48, 109.20, 69.46, 41.20, 37.75, 30.87, 27.69, 26.72, 21.18. HRMS (TOF-MS) (*m*/*z*): [M + H] calculated for C_15_H_20_O_4:_ 264.32; found 264.13. FT-IR: 1692 cm^–1^ (C=O ester).

### Synthesis of Perillyl Itaconate Containing Diluent

The reagents were mixed in the presence of the respective reactive diluent (perillyl alcohol, limonene, or linalool) in an equimolar amount. For example, itaconic anhydride (15.23 g, 135.88 mmol), perillyl alcohol (20.68 g, 135.88 mmol), and limonene (18.51 g, 135.88 mmol) were mixed in a one-neck round-bottom flask. The mixture was warmed to 50 °C and Novozym 435 was added (1.36 g, 2.5 wt % of total reagents). The reaction mixture was left to run overnight, and PerIt/Lim was recovered after enzyme filtration.

#### PerIt/Lim

^1^H NMR (400 MHz, CDCl_3_) δ 6.42 (dd, *J* = 34.5, 1.0 Hz, 1H), 5.91–5.70 (m, 2H), 5.40 (dqd, *J* = 5.1, 2.4, 1.6 Hz, 1H), 4.71 (dq, J = 6.8, 1.4 Hz, 4H), 4.53 (d, J = 28.8 Hz, 2H), 3.43–3.31 (m, 2H), 2.28–1.37 (m, 23H). ^13^C NMR (101 MHz, CDCl_3_) δ: 171.49, 170.57, 140.41, 149.71, 133.89, 133.41, 132.50, 130.88, 126.19, 120.77, 108.91, 108.49, 69.16, 41.21, 40.91, 37.45, 30.93, 30.72, 30.58, 28.04, 27.39, 26.43, 23.60, 20.95, 20.88. FT-IR: 1694 cm^–1^ (C=O ester) (42.4 g, 58% yield of PerIt).

#### PerIt/PA

^1^H NMR (400 MHz, CDCl_3_) δ 6.46–6.32 (m, 1H), 5.85–5.68 (m, 3H), 4.75–4.68 (m, 4H), 4.59–4.45 (d, *J* = 21.4 Hz, 2H), 4.01 (dq, *J* = 3.1, 1.1 Hz, 2H), 3.37 (dd, *J* = 14.3, 1.0 Hz, 2H), 2.20–1.42 (m, 20H). ^13^C NMR (101 MHz, CDCl_3_) δ: 171.08, 170.61, 149.90, 149.71, 137.21, 133.46, 132.49, 130.69, 126.16, 122.75, 108.89, 108.79, 69.29, 67.36, 41.24, 40.90, 37.48, 30.57, 30.52, 27.58, 27.39, 26.41, 26.22, 20.91, 20.87. FT-IR: 1698 cm^–1^ (C=O ester) (14.7 g of PerIt/PA, 61% yield of PerIt).

#### PerIt/Lin

^1^H NMR (400 MHz, CDCl_3_) δ 6.42–6.28 (dd, *J* = 32.3, 1.0 Hz, 1H), 5.84 (dd, *J* = 17.3, 10.8 Hz, 1H), 5.78–5.63 (m, 2H), 5.21–4.95 (m, 3H), 4.68–4.60 (m, 2H), 4.51–4.38 (m, 2H), 3.31 (dd, *J* = 14.9, 1.0 Hz, 2H), 2.14–1.36 (m, 20H), 1.21 (s, 3H). ^13^C NMR (101 MHz, CDCl_3_) δ: 171.20, 170.59, 149.71, 145.02, 133.45, 132.50, 132.10, 130.73, 126.16, 124.41, 111.89, 108.90, 73.79, 69.14, 42.14, 40.91, 37.47, 30.57, 27.92, 27.39, 26.42, 25.82, 22.91, 20.87, 17.82. FT-IR: 1698 cm^–1^ (C=O ester) (13.5 g of PerIt/Lin, 51% yield of PerIt).

### 2D-Thermosets Preparation for Thermomechanical Characterization

Resins were prepared by adding perillyl itaconate PerIt (bulk or containing diluents), trimethylolpropane tris(3-mercaptopropionate) 3T (thiol:ene ratios of 1:1, 0.66:1 or 0.33:1), and Irgacure 819 (1.5 or 5 wt %) into 7 mL glass vials equipped with magnetic stir bars. For a 1:1 thiol:ene ratio formulation, for example, perillyl itaconate (0.6 g, 2.26 mmol, 1 equiv) and trimethylolpropane tris(3-mercaptopropionate) (0.9 g, 2.26 mmol, 1 equiv) were added to a vial containing Irgacure 819 (0.023 g, 1.5 wt %). For formulations containing diluents, the thiol:ene ratios were calculated considering the double bonds from both the diluent and perillyl itaconate. The mixtures were stirred at 50 °C until all of the reagents were miscible and placed on rectangular glass slides, which were exposed to a UV light source (Omnicure S1500 fitted with a fiber optic cable, 405 nm) for 1 h. The cured films (∼0.7 mm thickness) were postcured in an oven at 120 °C for 16 h to ensure full network cross-linking before thermomechanical characterization.

### Resin Preparation for 3D Printing

Resins were prepared by mixing PI, 3T, and I819 with the respective additives. For example, PerIt/Lim:3T 1:1 resin was prepared by mixing PerIt:Lim monomer (10 g, 55.24 mmol), 3T (17.14 g, 43.00 mmol), I819 (1.36 g, 5 wt % to monomers), butylated hydroxytoluene BHT (0.27 g, 1 wt % to monomers), and Sudan Red II (8.14 mg, 0.03 wt % to monomers). The resin was stirred at 50 °C until all reagents were miscible and was further used for printing.

## Results and Discussion

### Photoactive Ester Synthesis and Network Formation

**Figure 1:**
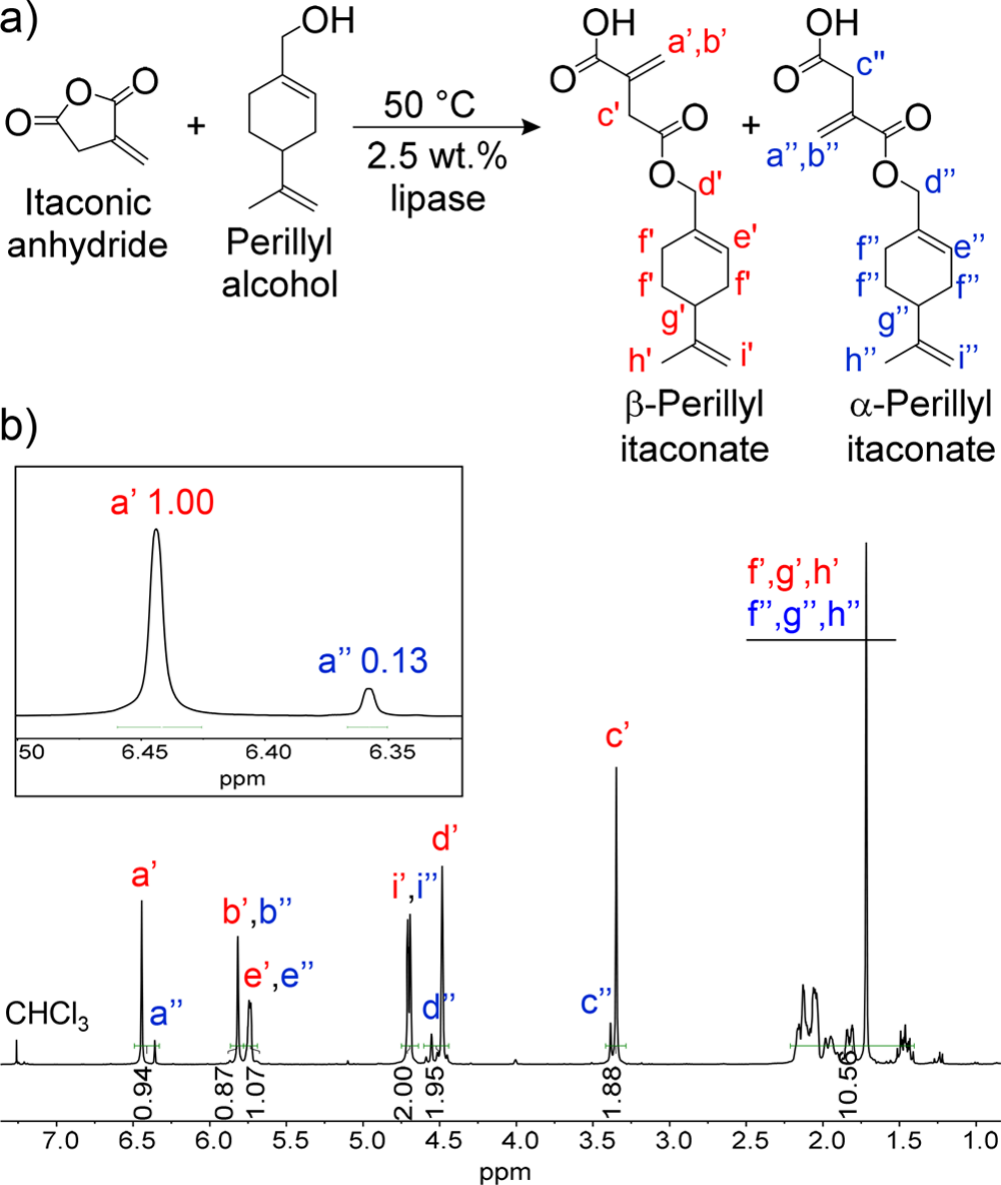
(a) 1-step synthesis of photoactive perillyl itaconate ester from the enzymatic ring-opening reaction of itaconic anhydride with perillyl alcohol. (b) ^1^H NMR spectrum of perillyl itaconate (300 MHz, 298 K, CDCl_3_).

In order to obtain a biobased alkene functional ester, itaconic anhydride was esterified with sustainably sourced perillyl alcohol in the presence of a commercial lipase (Novozym 435) to form perillyl itaconate (PerIt) in bulk. Biological-derived enzyme was selected to achieve a more sustainable pathway, as they can catalyze reactions under mild conditions and simple processes. Furthermore, studies have demonstrated the ability of Novozym 435 to be reused in several reaction cycles without losing its activity.^31^ The unsymmetrical anhydride structure led to the formation of α- and β-perillyl itaconate as two distinct addition products, with the β-adduct corresponding to 88 mol % of the final product. Monomer consumption over time was monitored by ^1^H NMR spectroscopy with conversions above 90% obtained after 8 h. The reaction could be easily scaled (up to 60 g) to afford a light-yellow waxy solid in high yield. Purification of a sample for initial proof of principle studies and characterization was possible using silica column purification (90:10 DCM:MeOH) (Figure 1, Figures S1, S2).

**Figure 2:**
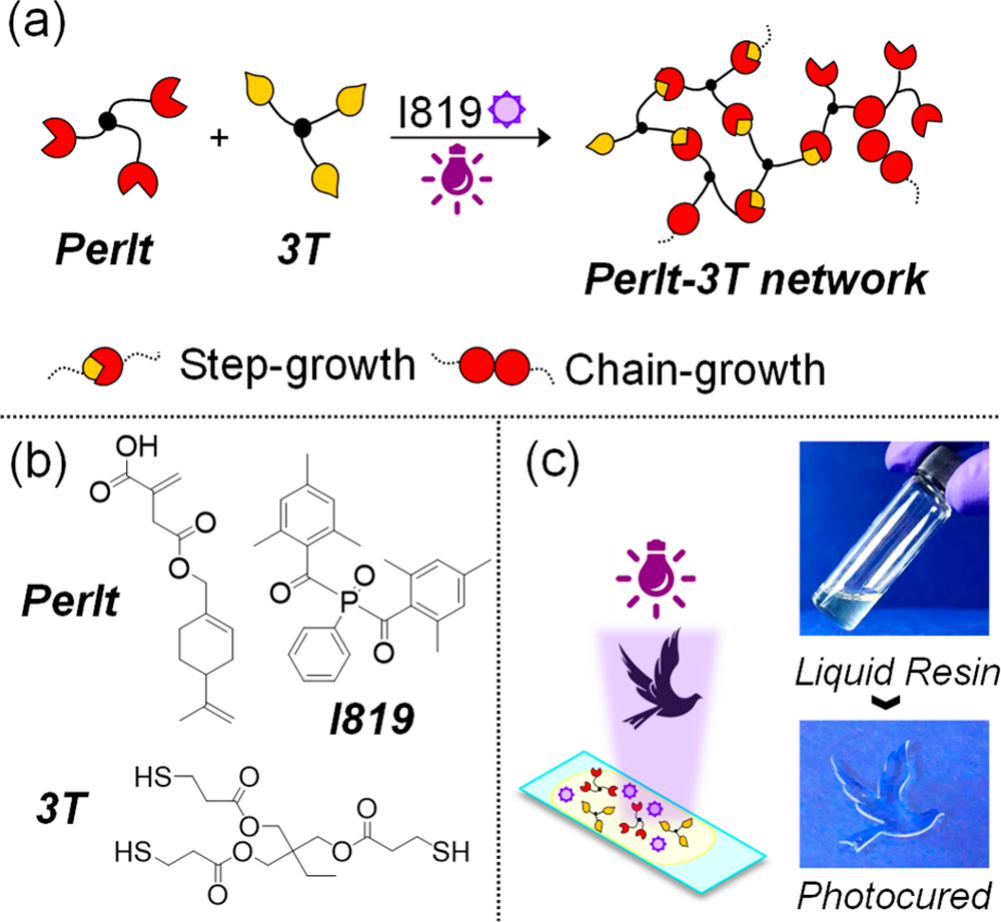
(a) Schematic depiction of network formation illustrating both step-growth and chain-growth polymerization. (b) Structure of photoactive monomer perillyl itaconate (PerIt), trimethylolpropane tris(3-mercaptoproprionate) (3T) and photoinitiator Irgacure 819 (I819). (c) Two-dimensional dove photocured in a DLP printer (405 nm, 225 W/m^2^ irradiance, 60 s of light irradiation).

Thiol–ene systems undergo step-growth polymerization and generally form uniform molecular networks with adjustable properties.^2,32^ In seeking to exploit the potential of PerIt to form photosets with a wide range of thermomechanical properties, thiol–ene polymerization of PerIt was performed using a multivalent thiol (3T) in the presence of the photoinitiator Irgacure 819 (I819) (Figure 2a,b). As such, different thiol:ene stoichiometries were utilized to investigate the effect of this variable on network properties. When an equimolar amount of PerIt and 3T (1:1) at 50 °C (in order to melt PerIt) was mixed with 1.5 wt % of photoinitiator, photopatterning could be performed using a DLP printer to form thin films with defined shape (Figure 2c). Notably, PerIt contains an activated acrylic-type double bond from the itaconate moiety as well as the less activated double bonds from the terpene in its structure. Hence, concomitant step-growth thiol–ene and thiol–acrylate reactions as well as chain-growth homopolymerization of the acrylic bonds will occur. In instance, networks with different properties, such as storage modulus and glass transition temperature, can be accessed by controlling the thiol stoichiometry and consequently the proportionality of the mechanisms of polymerization.^33^

In order to develop a more sustainable system where no laborious purification of the PerIt monomer is required, resins containing equimolar amount of thiol:ene were formulated with nonpurified PerIt (all reactions were quenched at approximately 96% conversion), thiol, and I819. In turn, tensile testing showed negligible difference on the mechanical properties when comparing networks formulated with purified and nonpurified PerIt; thereafter all resins in this work were formulated using the monomers readily after the reaction (enzyme filtration was required to separate the product from the catalyst) (Table S1).

**Figure 3:**
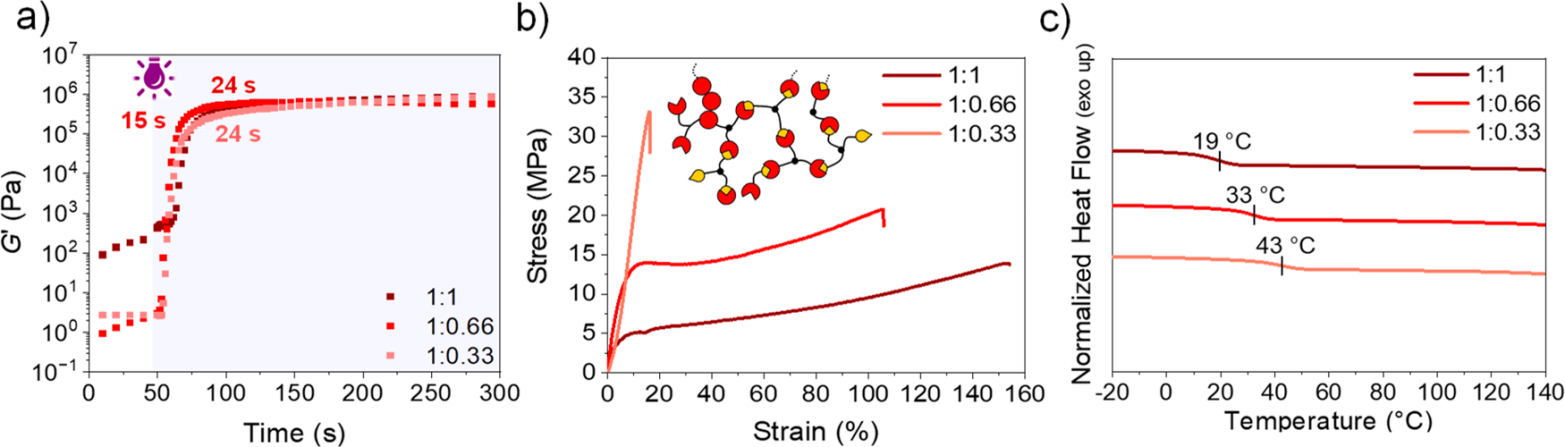
Thermomechanical characterization of PerIt:3T networks at different thiol:ene ratios and 5 wt % of I819. (a) Photorheology of mixtures over 300 s under oscillatory shear at room temperature. (b) Uniaxial tensile testing of networks at room temperature. (c) DSC thermograms of the second heating cycle from −20 to 140 °C.

With the purpose of expanding the material scope, the ratio of double bonds to thiol was decreased (from 1:1 to 1:0.33) to change cross-linking density and evaluate the stoichiometry effect on the thermomechanical properties. Gelation kinetics were investigated using real-time photorheology by monitoring the evolution of storage modulus *G*′ over time. Initially, resins were prepared using 1.5 wt % of I819 in their formulation; however, photocuring times from ∼1 to 3.7 min were found (see Figure S3). In an attempt to design resins for rapid photocuring applications, the mixtures were formulated using 5 wt % of I819 (Figure 3). In this case, an increase in *G*′ was observed for all samples after light irradiation, and a plateau was reached after 100–150 s, which indicates that complete curing had occurred. Thiol stoichiometry did not significantly influence gelation time and while 15 s were required to fully cure PerIt:3T 1:0.66 network, no differences were observed for concentrations of 1:1 and 1:0.33 (24 s) (Figure 3a).

FT-IR spectroscopy showed the disappearance of absorption peaks corresponding to allyl groups (∼1630 cm^–1^) after resin photoirradiation, confirming qualitative reduction of the functional groups under the investigated conditions (Figures S4, S5). 2D-thermosets were photocured on glass slides (∼ 1.5 mm thickness) and postcured overnight at 120 °C prior to mechanical testing (Figure 3b, Figure S6 for PerIt:3T with 1.5 wt % of I819). The formulation containing the lowest amount of thiol (PerIt:3T 1:0.33) demonstrated brittle behavior, with high ultimate tensile strength (UTS = 30.5 MPa) but low strain at break (15%). At higher levels of thiol monomer, more elastic materials with higher elongations at break (97 and 151% for 1:0.66 and 1:1, respectively) resulted. This observation is in accordance with a previous report from Bowman and co-workers, where the effect of thiol concentration was investigated in thiol–acrylate systems in which brittle materials resulted when monoacrylates/diacrylates were photocured with no thiol, however softer networks were achieved when thiol was added into the system.^34^

The thermal properties of the photocured networks were accessed by using differential scanning calorimetry (DSC). As expected, the prepared networks were all amorphous with glass transition temperature (*T*_g_) in a similar range or above ambient environments (19–43 °C). Also, the *T*_g_ increased by lowering the amount of thiol in the photoset composition, as higher concentrations of thioether bonds provide more flexibility and homogeneity to the network (Figure 3c, Figure S7 for PerIt:3T with 1.5 wt % of I819). This relation between thioether concentration and network flexibility is in accordance with previous reports, where the thermal properties of ternary thiol–ene/acrylate photopolymers were investigated.^35,36^

### Ester-Containing Diluents and Network Formation

**Figure 4:**
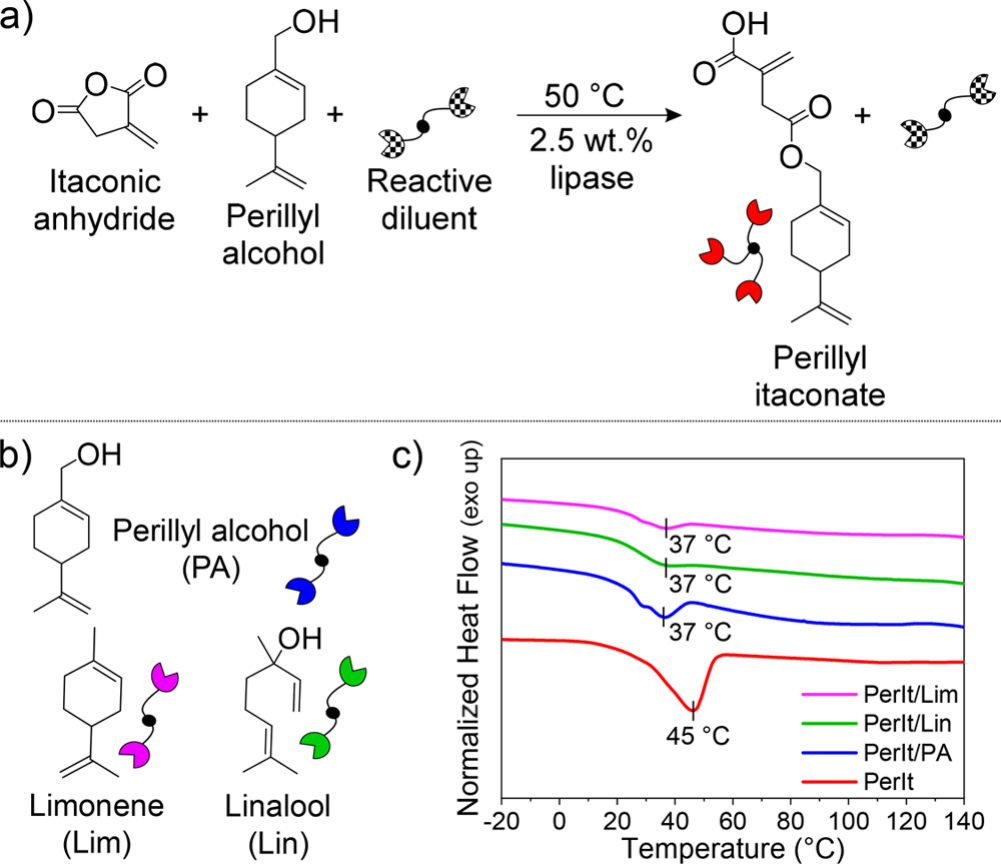
(a) Perillyl itaconate synthesis with reactive diluent addition. (b) Structure of the terpenes used as reactive diluents. (c) DSC thermograms of final monomers mixtures: second heating cycle from −20 to 40 °C.

Viscosity is a governing factor when designing new photocurable liquid formulations, as processability can be restricted at high viscosity ranges. A variety of biobased reactive diluents (RDs) have been used to address this limitation, with multifunctional (meth)acrylate-based monomers being prime examples.^37,38^ In addition to resin thinning effects, RDs can positively affect the mechanical properties of the final networks.^39^ In order to overcome the need to process resins at elevated temperatures, we added biosourced RDs during the synthesis of PerIt, thus presenting a simple solution to their dual use as biobased solvents and resin diluents (Figure 4a,b). On account of their reactivity, we envisaged that adding functional terpenes into the formulations would impact not only the physical characteristics of the resins but also the thermomechanical properties of the final photosets. Reactions were carried out using an equimolar amount of anhydride, perillyl alcohol, and reactive diluent, with excess of perillyl alcohol for PerIt/PA synthesis (see Figures S11–S13, Figures S33–S35). No conversion to linalyl itaconate was observed when linalool was used as a reactive diluent, most likely due to reduced activity of the sterically encumbered tertiary alcohol (Figures S22–S24). Overall, all reactions were stopped after overnight reaction when conversions were around 90–95%.

**Figure 5:**
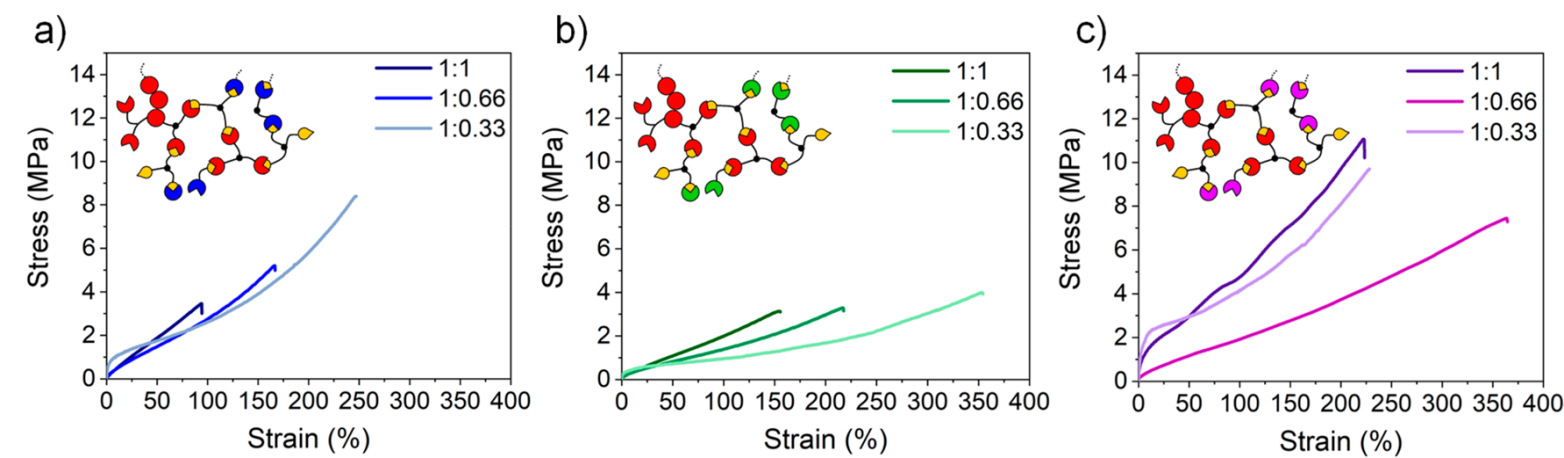
Uniaxial tensile testing of PerIt/diluents:3T networks at different thiol:ene ratios and 5 wt % of I819 at room temperature. (a) Perillyl itaconate (PerIt)/perillyl alcohol (PA):3T. (b) PerIt/linalool (Li):3T. (c) PerIt/Limonene (Lim):3T.

Addition of the diluents decreased the melting point of PerIt from 45 to 37 °C (for PerIt/Lim, PerIt/Lin and PerIt/PA); however all products slowly solidified at room temperature to give a two-phase mixture of waxy solid and viscous liquid (Figure 4c). When 3-arm thiol was added into the formulations, the resins remained miscible at room temperature. FT-IR spectroscopy showed the disappearance of absorption peaks corresponding to allyl groups (∼1630 cm^–1^) after resin photoirradiation, indicating qualitative reduction of the functional groups under the investigated conditions (Figures S14–15, S25–26, S36–37). Gel fractions ranging from 72 to 100% were also a great indicator of the high cross-linked nature of the formed networks (Table S2). Tensile mechanical testing (Figure 5) for photosets with different compositions was performed to elucidate the effect of the reactive diluents on the mechanical properties. As anticipated, the mechanical performance could be tailored by controlling the reactive diluent type, thiol stoichiometry, and photoinitiator concentration (Figures S16, S27, and S38 for formulations with 1.5 wt % of I819). In general, photosets containing diluents in their composition presented higher elongation at break (up to 367%) than the previously synthesized PerIt:3T (up to 151%), indicating moderate to high flexibility of these materials. Following a similar trend to PerIt:3T networks, PerIt/PA:3T containing lower amount of thiol (1:0.33) presented the higher UTS (7.7 MPa) for networks containing perillyl alcohol as diluent but differed from PerIt:3T in terms of elongation capability (Figure 5a). For PerIt/Lin:3T and PerIt/Lim:3T, however, UTS did not follow a clear trend when the thiol concentration was investigated. While both systems displayed elastic behavior, stronger materials were found for formulations containing limonene (UTS = 6.3–10.6 MPa, ε_break_ = 220–321%) when compared to linalool (UTS = 3.1–4.1 MPa, ε_break_ = 158–353%). While Young’s modulus (*E*) was limited to 0.4–0.5 MPa (for linalool-, nerol-, and geraniol-based photosets) and 43.8 MPa (for limonene-based photosets) in our previous report,^29^ in here we disclosed materials with *E* varying from 3.6 to 358 MPa. PerIt-based networks presented highly elastic and flexible features and were comparable to commercial resins, such as Photocentric Flexible UV160TR, Formlabs Elastic 50A, and Formlabs Flexible 80A (Table S3).^40−42^ In addition, the designed photosets demonstrated similar or superior mechanical properties when compared to other reported biobased thiol–ene networks.^43−47^ For example, Porwal et al. reported renewable networks from triallyl levoglucosan and multifunctional thiols with *E* and UTS varying from 3.3 to 14.5 MPa and 1.0 to 2.7 MPa, respectively.^46^

The glass transition temperature of the new photosets was determined by DSC, and a decrease in *T*_g_ was observed for thermosets containing diluents. For formulations containing 0.33 equiv of thiol and 5 wt % of I819, for example, the *T*_g_ reduced from 43 °C in PerIt:3T networks to around 20 °C when PA, Lin, or Lim were used as reactive diluents (Table S2, Figure 3c, Figure S7, S17–18, S28–29, S39–40). Overall, all thermosets displayed high thermal stability with decomposition temperatures (*T*_d,5%_) ranging from 190 to 236 °C, with higher thiol contents impacting positively *T*_d,5%._ (Table S2, Figures S8–9, S19–20, S30–31, S41–42). Dynamic mechanical analysis (DMA) revealed large rubbery plateaus for the formed networks, with a significant decrease in storage modulus *E*′ above the glass transition temperatures (Figure S10, S21, S32 and S43). For PerIt/Lim:3T networks, higher rubbery plateau modulus *E*′_rubbery_ were found at greater concentrations of thiol (1135 MPa for 1:1 versus 279 MPa for 1:0.33), with cross-linked density ranging from 36.5 to 7.6 when lowering thiol content. However, this was not observed for the other sets of materials, where higher *E*′_rubbery_ values were observed at lower concentrations of thiol (Table S4). We hypothesize that this could be related to the higher probabilities of chain-growth reactions at lower thiol concentrations, leading to chain entanglement that cannot be differentiated from cross-links, and as the *E*′_rubbery_ is a function of both, the calculation renders an apparent increase in cross-link density. Previous work on ternary thiol–ene/acrylate resins showed the decrease of *E*′_rubbery_ and *T*_*g*_ by increasing the thiol concentration in resin formulations, as the step-growth reactions result in more homogeneous networks.^35^ In addition to its versatile mechanical performance, the developed system also offers an avenue toward degradable networks on account of the functional ester linkages from PerIt and 3T. Previously reported thiol–ene networks containing ester functionalities showed susceptibility to hydrolytic degradation, with carboxylic acids and other small molecules as degradation products.^46,48^

### Translation of Biobased Resin to Additive Manufacturing

3D printing has been at the forefront of photoreactive resin research owing to its versatility and ability to form structures with high precision and at low cost. In a typical DLP process, a tank/vat containing a liquid resin is photoirradiated to cross-link successive layers in accordance with a 3D computer assisted design (CAD) digital model. Printing resolution is intrinsically related to resin viscosity, curing time and light absorption, and reactive diluents and photoinhibitors are often used to print objects with high-fidelity to their CAD models.^2,49^ We envisaged that self-supporting structures would be achievable using the PerIt/Lim:3T system due to its attractive mechanical features, with moderate elongation and the ability to support high strength before failure.

**Figure 6:**
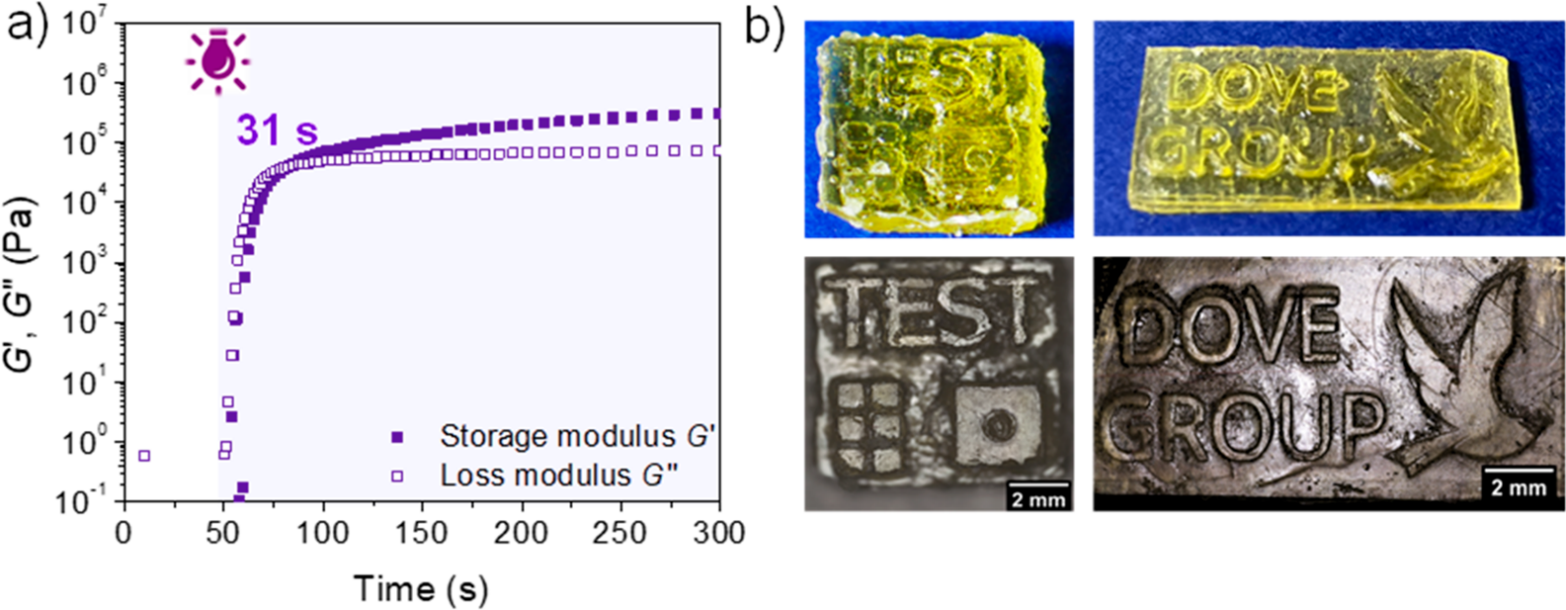
3D printing of PerIt/Lim:3T resin containing 1 wt % of BHT and 0.03 wt % of Sudan Red II. (a) Photorheology over 300 s under oscillatory shear at room temperature. (b) 3D printed parts containing test squares and Dove logo. Printing conditions: 50 μm and 120 s cure/layer.

Photorheology of PerIt/Lim:3T 1:1 revealed faster gelation kinetics (25 s) compared to PerIt/Lim:3T 1:0.66 and 1:0.33 (33 and 78 s, respectively) when 5 wt % of I819 was added into the formulation (Figure S45). The printing was performed on a DLP printer with a λ = 405 nm light source and 225 W/m^2^ irradiance. The 3D CAD model of a square base (0.95 cm × 0.95 cm, 42 layers, 50 μm per layer) containing text and inner squares/features was selected to access lateral (*x*, *y*) and vertical (*z*) printing resolution. Initial attempts were focused on PerIt/Lim:3T 1:1 formulation, with light exposure of 45, 60, 70, or 90 s per layer, but only squares with poor resolution and low fidelity, as compared to the CAD model, were formed. In order to prevent premature gelation in the resin tank, BHT was added into the formulation as a radical inhibitor (1 wt %) and the light exposure time was set to 90 s per layer. While the features could be printed according to the CAD model, the structure showed poor *z-*resolution (Figure S46). Light absorbers/photoinhibitors are commonly used to reduce light penetration depth, allowing the photocuring of thinner layers and consequently better *z*-resolution. On account of this, Sudan Red II was added at a concentration of 0.03 wt % into the PerIt/Lim:3T 1:1/1 wt % of BHT formulation. The new resin formulation revealed fast photocuring (31 s), viscosity of 60 mPa·s, and a cure depth of 110 μm after 110 s of light irradiation using the DLP printer (Figure 6a, Figures S47, S48). This resin formulation was prepared on a 30 g scale and remained stable for weeks when stored in the absence of light. After initial tests (Figure S50), an optimal printing condition was found when the resin was printed using 120 s of cure per layer to give 3D printed parts with good resolution and fidelity to the CAD models (Figure 6b). It is noteworthy that longer light exposure (120 s) was required when using the DLP printer compared to photorheology performed in the rheometer (31 s), and this is related to layer thickness (0.02 and 0.05 mm for rheometer and printer, respectively) and the different light intensities of these instruments.

To increase the renewable content of the system, we also formulated a resin containing a lower thiol concentration (1:0.33). In turn, by decreasing the thiol concentration from 1:1 to 1:0.33, the biobased content was raised from 36.6 to 63.6 wt %, respectively (Table S2). As expected, lowering the thiol concentration led to increased resin viscosity (120 mPa·s compared to 60 mPA·s for the PerIt/Lim:3T 1:1 resin). Thinner layers (90 μm after 110 s in the DLP) were photocured to overcome the longer curing time required for this resin (Figures S47, S49). Given the competitive step-growth and chain-growth polymerizations, we anticipated that the printing parameters would change when lowering the thiol content. Indeed, higher concentrations of photoinitiator (7 wt %) and inhibitors (2 wt % of BHT and 0.06 wt % of Sudan Red II) were required to print the square test (Figure S51). Despite the ability to print, the resolution of the 3D parts was inferior when compared with the resin containing higher thiol contents. This could, however, be further optimized by changing the type and concentration of additives in the formulation.

## Conclusions

A library of renewable photosets with adjustable thermomechanical properties were prepared via thiol–ene reactions for rapid photocuring applications. The resin systems were based on natural-derived terpenes/terpenoids and itaconic acid, and synthetic procedures were carried out under mild conditions and in the absence of organic solvents. In their majority, the designed photosets showed elastic and flexible behavior with comparable properties to 3D networks prepared from commercial resins. However, networks based on step-growth polymerization degrade and leave behind small molecule byproducts that cannot be recured. Future work will focus on designing renewable covalent adaptable networks that can be recycled back into their original dynamic monomers, enabling the formation of a closed-loop system. Nevertheless, this platform offers a simple, scalable, and environmentally friendly route that can potentially replace nonrenewable and nondegradable networks.

## Data Availability

All data are available in the manuscript or the Supporting Information.

## Supporting Information Available

^1^H NMR, ^13^C NMR, FT-IR, rheology, tensile, DSC, TGA, DMA, and gel fraction characterization (PDF)
