# Supplementary material for: Improving the Performance of Photoactive Terpene-Based Resin Formulations for Light-Based Additive Manufacturing
Source: ACS Sustain Chem Eng. 2024 Apr 24;12(18):6904–12. doi: 10.1021/acssuschemeng.3c08191 (PMC11077580; doi:10.1021/acssuschemeng.3c08191)
Supplement: Supplementary file 1 — sc3c08191_si_001.pdf [file sc3c08191_si_001.pdf]

Supporting Information

Improving the performance of photoactive terpenes in resin formulations for additive manufacturing

Viviane Chiaradia,[1] Elena Pensa,[1] Thiago O. Machado,[1] Andrew P. Dove[1*]

School of Chemistry, University of Birmingham, Edgbaston, Birmingham, B15 2TT, United Kingdom.

* Corresponding author. Email: a.dove@bham.ac.uk

Contents:

Fig. S1 ^{13}C NMR Spectrum of PerIt ester - 101 MHz, 298 K, CDCl3. .... S4

Fig. S2 FT-IR spectra of PerIt monomer. S4

Fig. S3 Photorheology of PerIt:3T at different thiol:ene ratios using 1.5 wt.% of photoinitiator under oscillatory shear at room temperature. S5

Fig. S4 FT-IR spectra of PerIt:3T networks (1.5 wt.% I819). S5

Fig. S5 FT-IR spectra of PerIt:3T networks (5 wt.% I819). S6

Fig. S6 Uniaxial tensile testing of PerIt:3T networks at different thiol:ene ratios and 1.5 wt.% of I819 at room temperature. S6

Fig. S7 DSC thermograms of PerIt:3T networks at different thiol:ene ratios and 1.5 wt.% of I819, second heating cycle from -20 to 140 °C. S7

Fig. S8 TGA thermograms of PerIt:3T networks at different thiol:ene ratios and 1.5 wt.% of I819. .... S7

Fig. S9 TGA thermograms of PerIt:3T networks at different thiol:ene ratios and 5 wt.% of I819. .... S8

Fig. S10 DMA temperature sweep for PerIt:3T networks at different thiol:ene ratios and 5 wt.% of I819. .... S8

Fig. S11 ^{1}H NMR spectrum of perillyl itaconate/perillyl alcohol monomer (300 MHz, 298 K, $CDCl_3$). . S9

Fig. S12 ^{13}C NMR Spectrum of PerIt/PA monomer - 101 MHz, 298 K, $CDCl_3$. S10

Fig. S13 FT-IR spectra of PerIt/PA monomer. S10

Fig. S14 FT-IR spectra of PerIt/PA:3T networks (1.5 wt.% I819). S11

Fig. S15 FT-IR spectra of PerIt/PA:3T networks (5 wt.% I819). S11

Fig. S16 Uniaxial tensile testing of perillyl itaconate (PerIt)/perillyl alcohol (PA):3T networks. S12

Fig. S17 DSC thermograms of PerIt/PA:3T networks at different thiol:ene ratios and 1.5 wt.% of I819. Second heating cycle from -20 to 140 °C. S12

Fig. S18 DSC thermograms of PerIt/PA:3T networks at different thiol:ene ratios and 5 wt.% of I819. Second heating cycle from -20 to 140 °C. S13

Fig. S19 TGA thermograms of PerIt/PA:3T networks at different thiol:ene ratios and 1.5 wt.% of I819. .... S13

Fig. S20 TGA thermograms of PerIt/PA:3T networks at different thiol:ene ratios and 5 wt.% of I819. .... S14

Fig. S21 DMA temperature sweep for PerIt/PA:3T networks at different thiol:ene ratios and 5 wt.% of I819. .... S14

Fig. S22 ^{1}H NMR spectrum of perillyl itaconate/linalool monomer (300 MHz, 298 K, $CDCl_3$). S15

Fig. S23 ^{13}C NMR Spectrum of PerIt/Lin monomer - 101 MHz, 298 K, $CDCl_3$ S16

Fig. S24 FT-IR spectra of PerIt/Lin monomer. S16

Fig. S25 FT-IR spectra of PerIt/Lin:3T networks (1.5 wt.% I819). S17

Fig. S26 FT-IR spectra of PerIt/Lin:3T networks (5 wt.% I819). S17

Fig. S27 Uniaxial tensile testing of perillyl itaconate (PerIt)/linalool (Lin):3T networks. S18

Fig. S28 DSC thermograms of PerIt/Lin:3T networks at different thiol:ene ratios and 1.5 wt.% of I819. Second heating cycle from -20 to 140 °C. S18

Fig. S29 DSC thermograms of PerIt/Lin:3T networks at different thiol:ene ratios and 5 wt.% of I819. Second heating cycle from -20 to 140 °C. S19

Fig. S30 TGA thermograms of PerIt/Lin:3T networks at different thiol:ene ratios and 1.5 wt.% of I819. .... S19

Fig. S31 TGA thermograms of PerIt/Lin:3T networks at different thiol:ene ratios and 5 wt.% of I819. .... S20

Fig. S32 DMA temperature sweep for PerIt/Lin:3T networks at different thiol:ene ratios and 5 wt.% of I819. .... S20

Fig. S34 ^{13}C NMR Spectrum of PerIt/Lim monomer - 101 MHz, 298 K, $CDCl_3$. S22

Fig. S35 FT-IR spectra of PerIt/Lim monomer. S22

Fig. S36 FT-IR spectra of PerIt/Lim:3T networks (1.5 wt.% I819). S23

Fig. S37 FT-IR spectra of PerIt/Lim:3T networks (5 wt.% I819). S23

Fig. S38 Uniaxial tensile testing of perillyl itaconate (PerIt)/limonene (Lim):3T networks. S24

Fig. S39 DSC thermograms of PerIt/Lim:3T networks at different thiol:ene ratios and 1.5 wt.% of I819. Second heating cycle from -20 to 140 °C. S24

Fig. S40 DSC thermograms of PerIt/Lim:3T networks at different thiol:ene ratios and 5 wt.% of I819. Second heating cycle from -20 to 140 °C. S25

Fig. S41 TGA thermograms of PerIt/Lim:3T networks at different thiol:ene ratios and 1.5 wt.% of I819. .... S25

Fig. S42 TGA thermograms of PerIt/Lim:3T networks at different thiol:ene ratios and 5 wt.% of I819. .... S26

Fig. S43 DMA temperature sweep for PerIt/Lim:3T networks at different thiol:ene ratios and 5 wt.% of I819. .... S26

Fig. S44 Photorheology of PerIt/Lim:3T at different thiol:ene ratios using 1.5 wt.% of photoinitiator under oscillatory shear at room temperature. S27

Fig. S45 Photorheology of PerIt/Lim:3T at different thiol:ene ratios using 5 wt.% of photoinitiator under oscillatory shear at room temperature. S27

Fig. S46 (a) 3D CAD model of a square test used for precise DLP. (b) Photograph of 3D-printed part from PerIt/Lim-3T 1:1, 5 wt.% of I819 and 1 wt.% of BHT. Printing conditions: 50 µm and 90 s cure/layer. (C) Microscope image of the printed part........ S28

Fig. S47 PerIt/Lim:3T resins containing additives. (a) 5 wt.% of I819, 1 wt.% of BHT and 0.03 wt.% of Sudan Red II. (b) Resin viscosity between a shear rate of 1 to 100 s-1 for PerIt/Lim:3T 1:1 (5 wt.% of I819, 1 wt.% of BHT and 0.03 wt.% of Sudan Red II) and PerIt/Lim:3T 1:0.33 (7 wt.% of I819, 2 wt.% BHT and 0.06 wt.% of Sudan Red II)........ S28

Fig. S48 Cure depth versus exposure time for PerIt/Lim:3T 1:1 (5 wt.% of I819, 1 wt.% of BHT and 0.03 wt.% of Sudan Red II). S29

Fig. S49 Cure depth versus exposure time for PerIt/Lim:3T 1:0.33 (7 wt.% of I819, 2 wt.% BHT and 0.06 wt.% of Sudan Red II). S29

Fig. S50 (a) 3D-printed part from a resin containing PerIt/Lim-3T in 1:1 ratio, 5 wt.% of I819, 1 wt.% of BHT and 0.01 wt.% of Sudan Red II. Printing conditions: (A) 25 µm and 100 s cure/layer and (B) 50 µm and 110 s cure/layer........ S30

Fig. S51 (a) 3D-printed part from a resin containing PerIt/Lim-3T in 1:0.33 ratio, 7 wt.% of I819, 2 wt.% of BHT and 0.06 wt.% of Sudan Red II. Printing conditions: (A) 25 µm and 100 s cure/layer and (B) 50 µm and 110 s cure/layer. S30

Table S1. Comparative of mechanical properties of resins formulated with purified and non-purified PerIt. Formulation containing PerIt:3T 1:1 and 1.5 wt.% of I819........ S30

Table S2. Summary of thermomechanical data and gel fraction for all designed networks. S31

Table S3. Mechanical properties for commercial 3D printing resins. S31

Table S4. Summary of DMA data for networks designed with 5 wt.% of I819. .... S32

Perlt monomer

^{13}C NMR spectrum of Perlt monomer

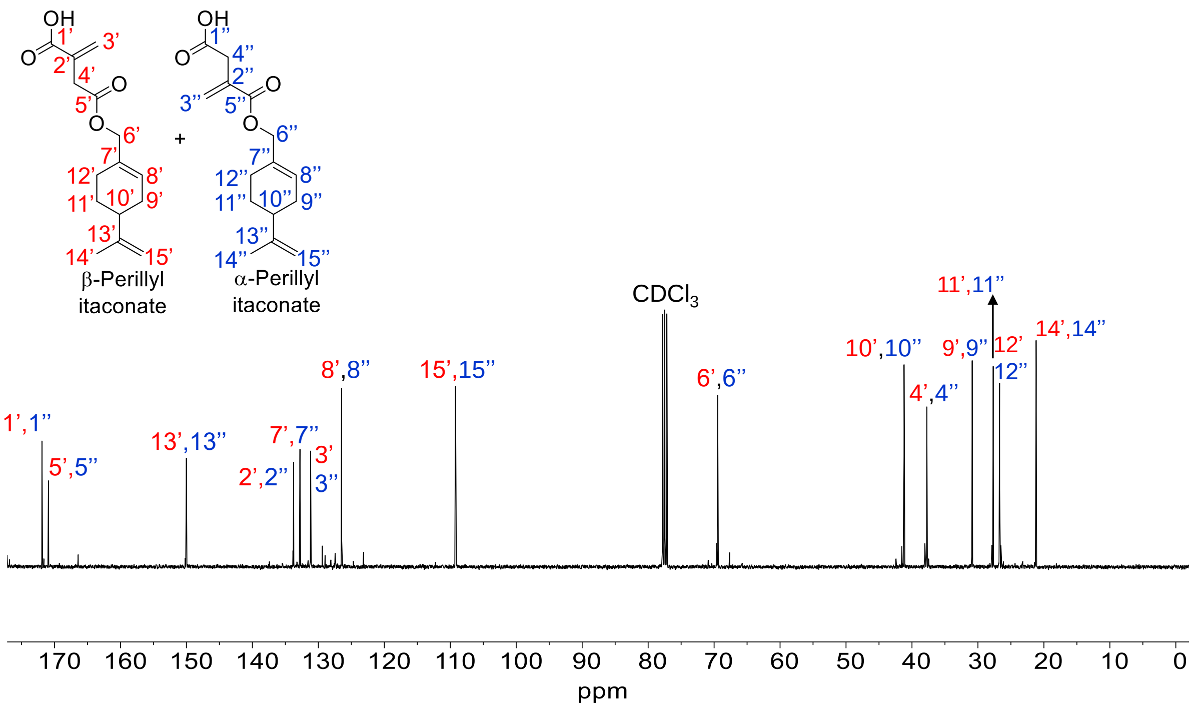

Fig. S1 ^{13}C NMR Spectrum of Perlt ester - 101 MHz, 298 K, CDCl3.

FT-IR spectra of Perlt monomer

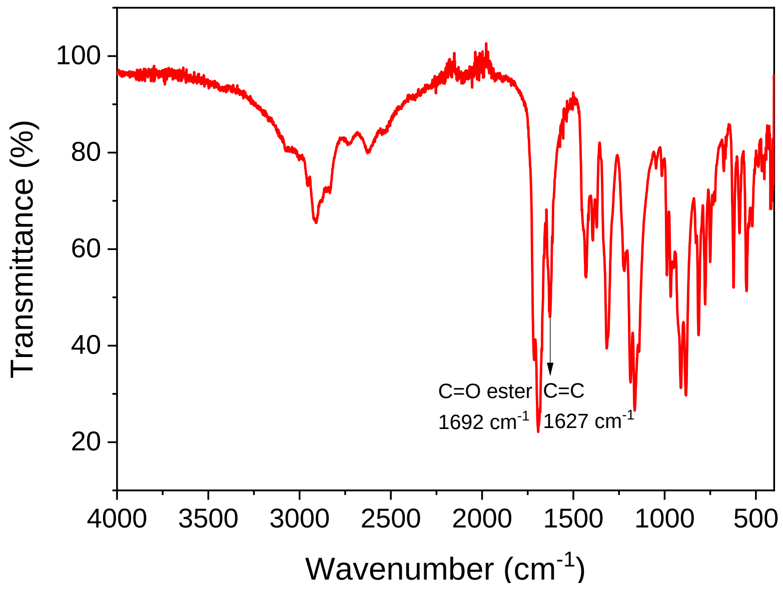

Fig. S2 FT-IR spectra of Perlt monomer.

Photoreology of PerIt:3T - 1.5 wt.% of I819

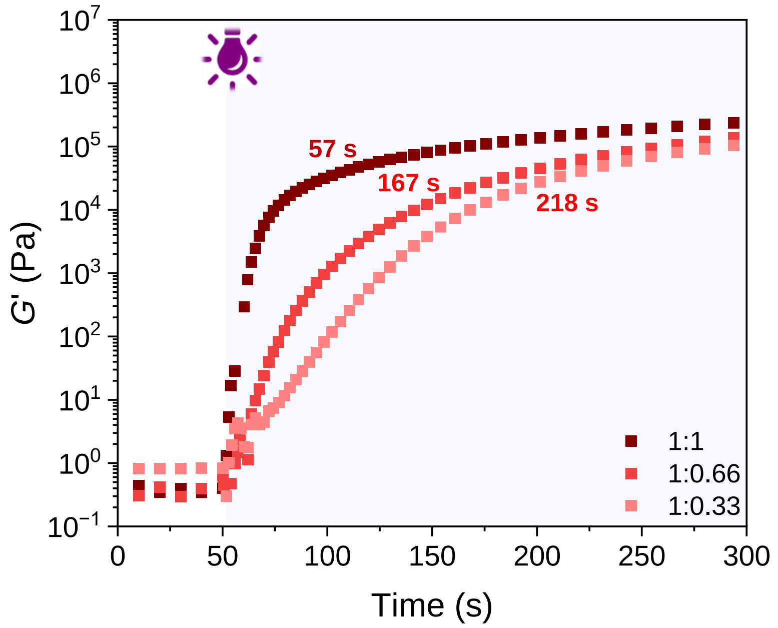

Fig. S3 Photorheology of PerIt:3T at different thiol:ene ratios using 1.5 wt.% of photoinitiator under oscillatory shear at room temperature.

FT-IR spectra of PerIt:3T photosets - 1.5 wt.% I819

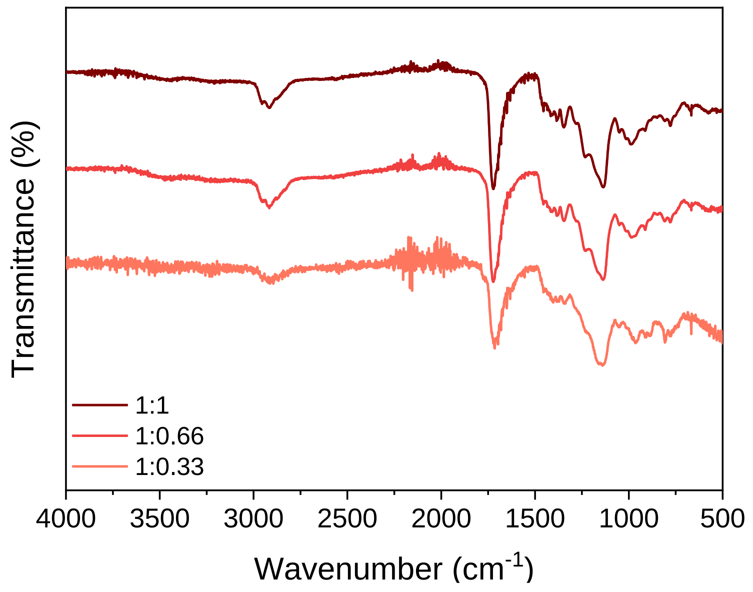

Fig. S4 FT-IR spectra of PerIt:3T networks (1.5 wt.% I819).

FT-IR spectra of PerIt:3T photosets - 5 wt.% I819

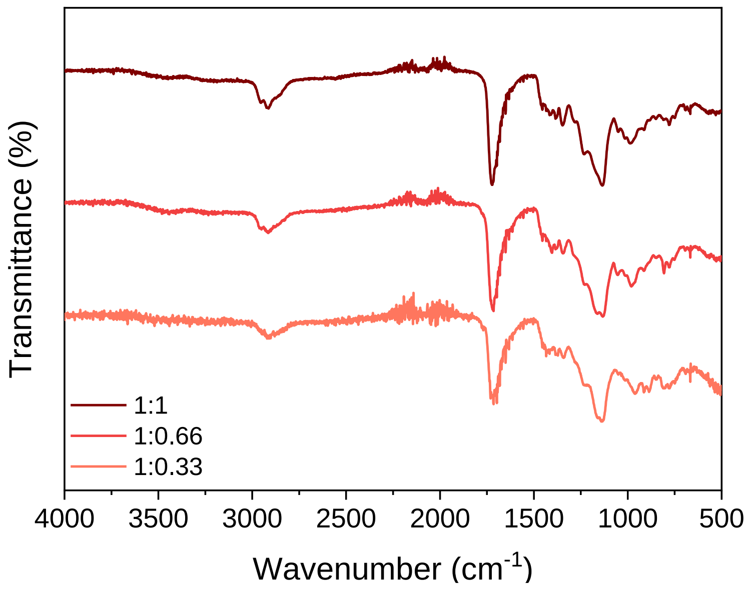

Fig. S5 FT-IR spectra of PerIt:3T networks (5 wt.% I819).

Tensile testing of PerIt:3T photosets - 1.5 wt.% of I819

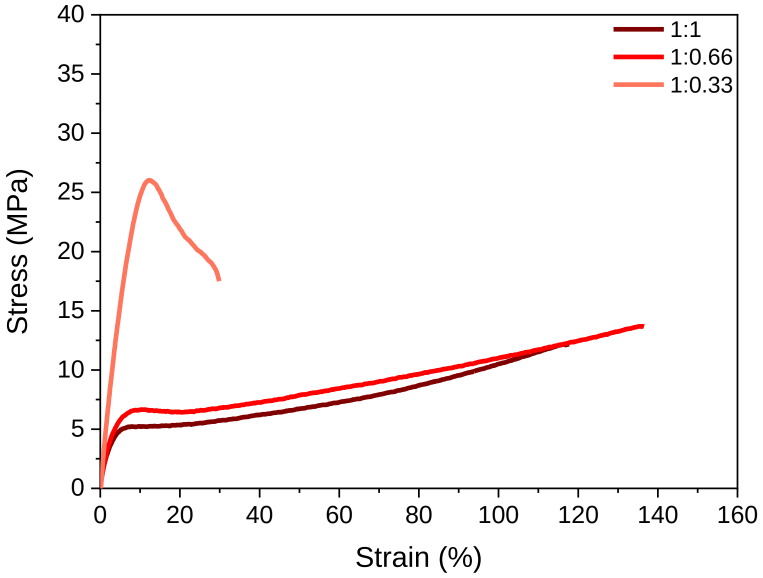

Fig. S6 Uniaxial tensile testing of PerIt:3T networks at different thiol:ene ratios and 1.5 wt.% of I819 at room temperature.

DSC data PerIt:3T photosets - 1.5 wt.% of I819

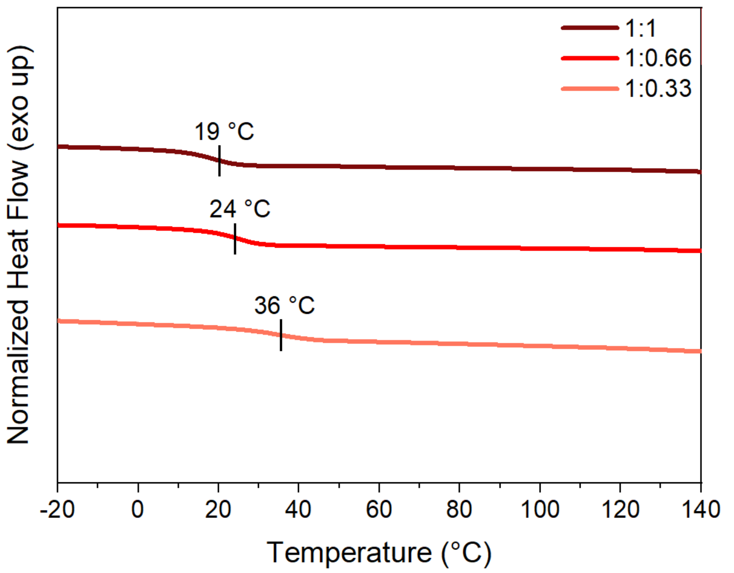

Fig. S7 DSC thermograms of PerIt:3T networks at different thiol:ene ratios and 1.5 wt.% of I819, second heating cycle from -20 to 140 °C.

TGA data PerIt:3T photosets - 1.5 wt.% of I819

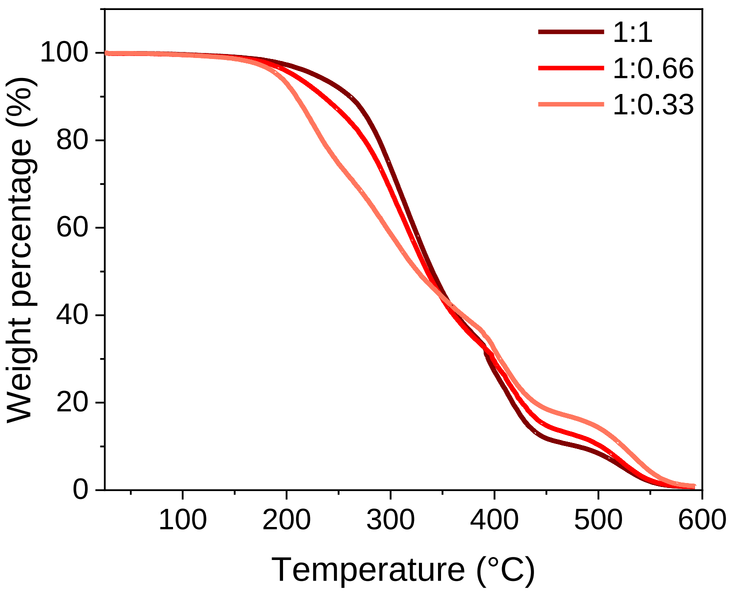

Fig. S8 TGA thermograms of PerIt:3T networks at different thiol:ene ratios and 1.5 wt.% of I819.

TGA data PerIt:3T photosets - 5 wt.% of I819

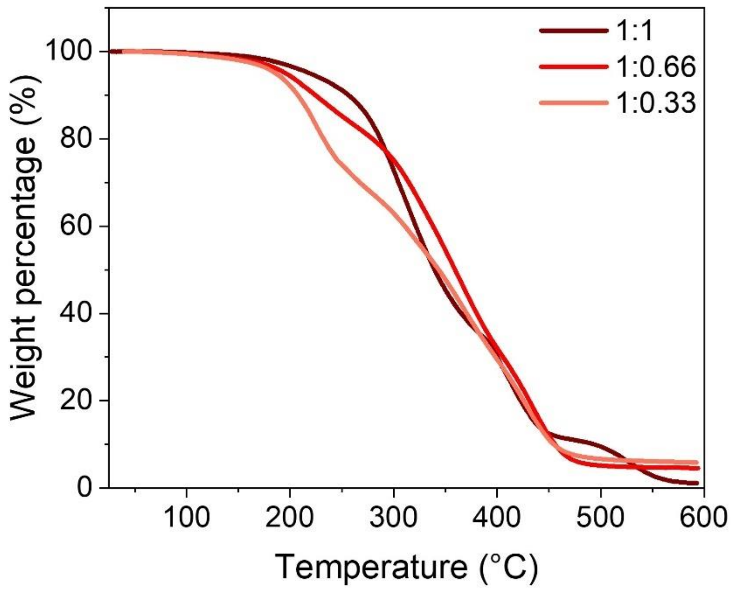

Fig. S9 TGA thermograms of PerIt:3T networks at different thiol:ene ratios and 5 wt.% of I819.

DMA data PerIt:3T photosets - 5 wt.% of I819

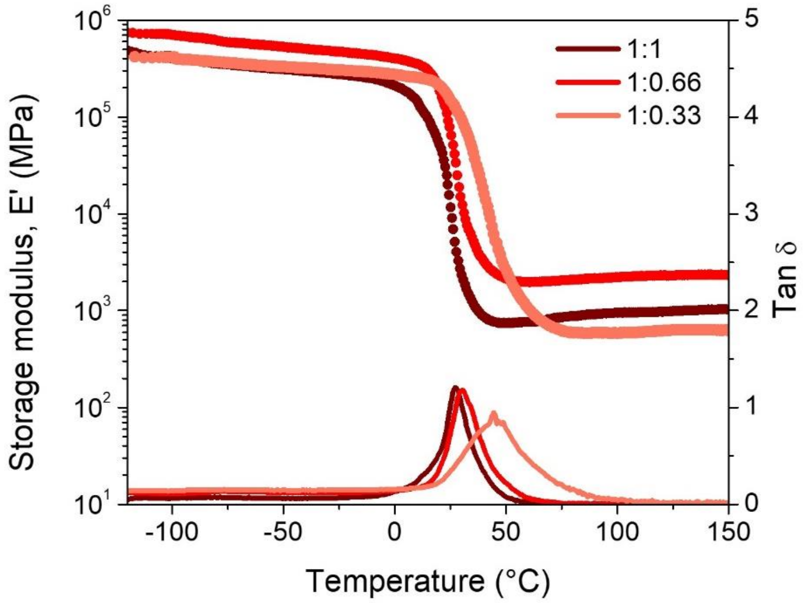

Fig. S10 DMA temperature sweep for PerIt:3T networks at different thiol:ene ratios and 5 wt.% of I819.

PerIt/PA monomer

<u>^{1}H NMR of PerIt/PA monomer</u>

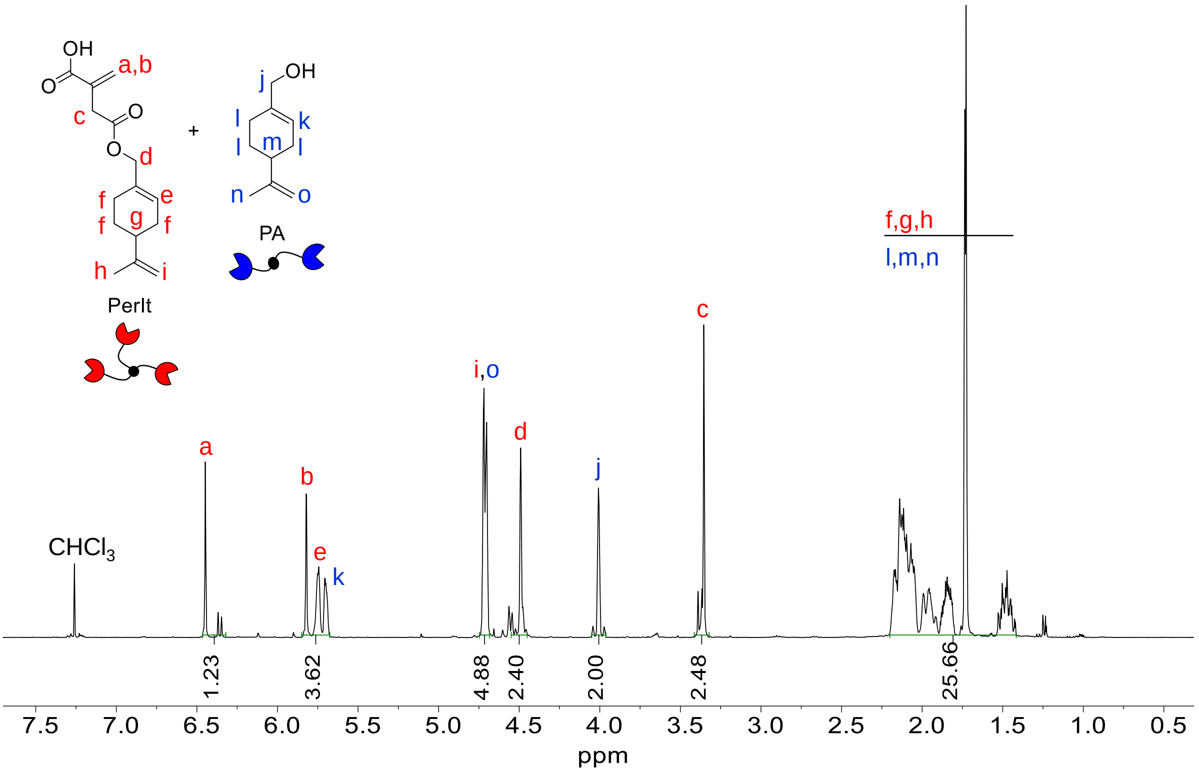

Fig. S11 ^{1}H NMR spectrum of perillyl itaconate/perillyl alcohol monomer (300 MHz, 298 K, $CDCl_3$).

^{13}C NMR of PerIt/PA monomer

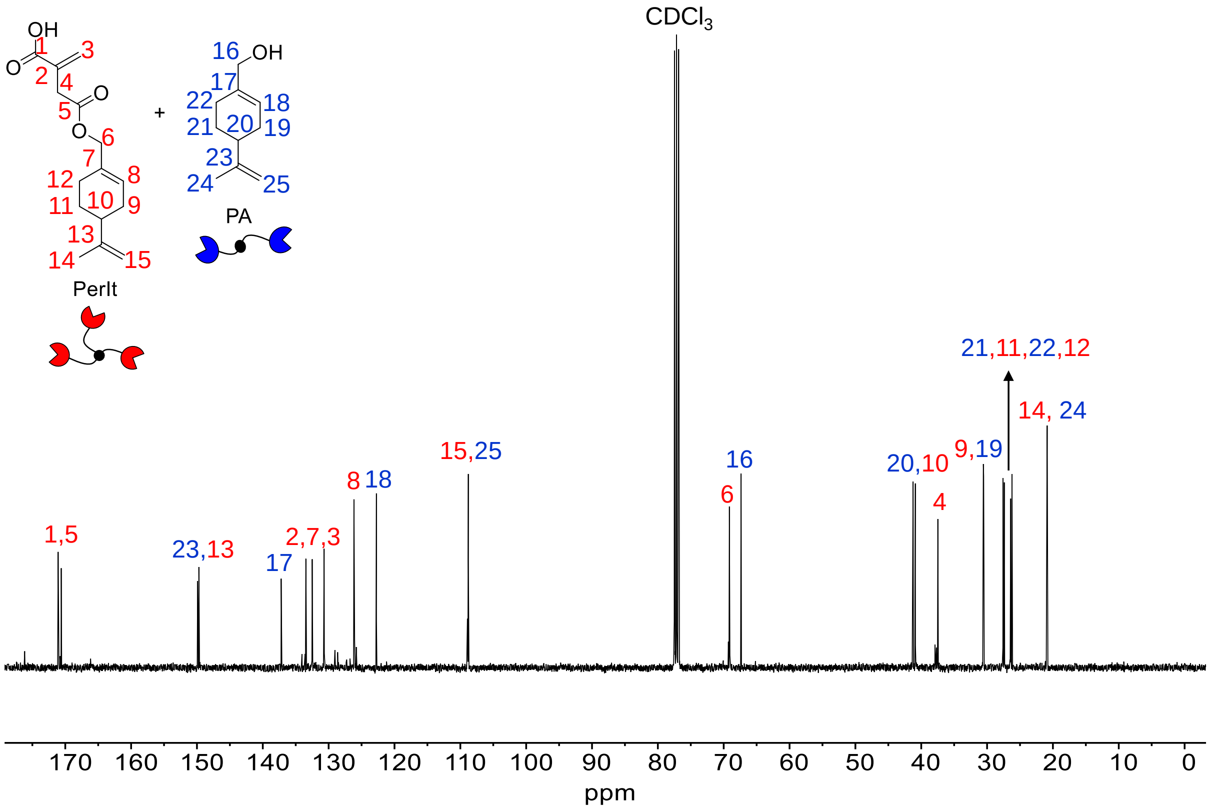

Fig. S12 ^{13}C NMR Spectrum of PerIt/PA monomer - 101 MHz, 298 K, $CDCl_3$.

5 FT-IR spectra of PerIt/PA monomer

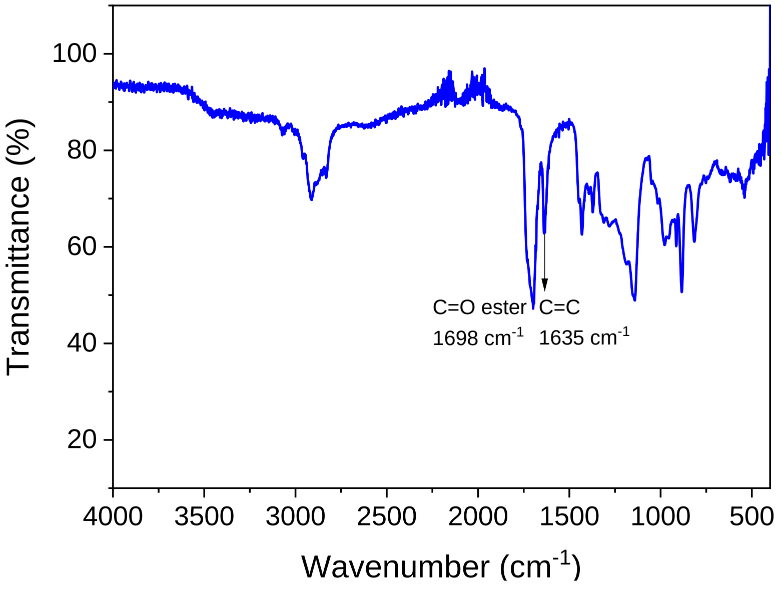

Fig. S13 FT-IR spectra of PerIt/PA monomer.

FT-IR spectra of PerIt/PA:3T photosets - 1.5 wt.% I819

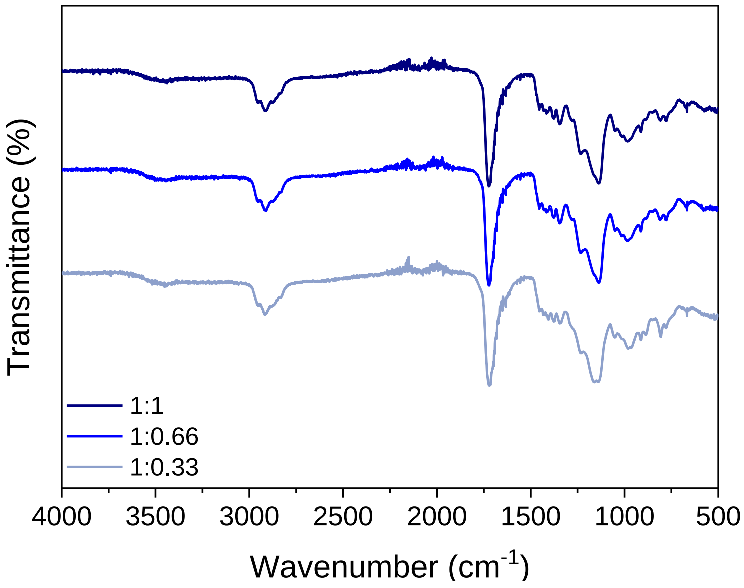

Fig. S14 FT-IR spectra of PerIt/PA:3T networks (1.5 wt.% I819).

FT-IR spectra of PerIt/PA:3T photosets - 5 wt.% I819

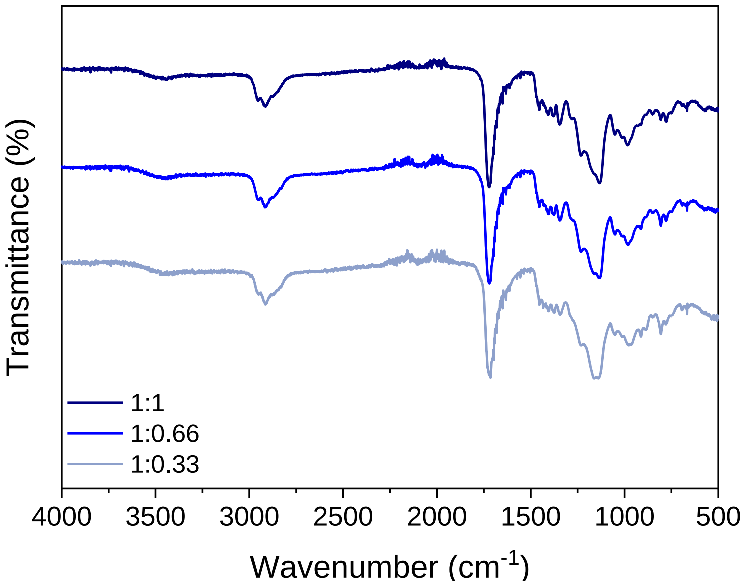

Fig. S15 FT-IR spectra of PerIt/PA:3T networks (5 wt.% I819).

Tensile testing of PerIt/PA:3T photosets - 1.5 wt.% of I819

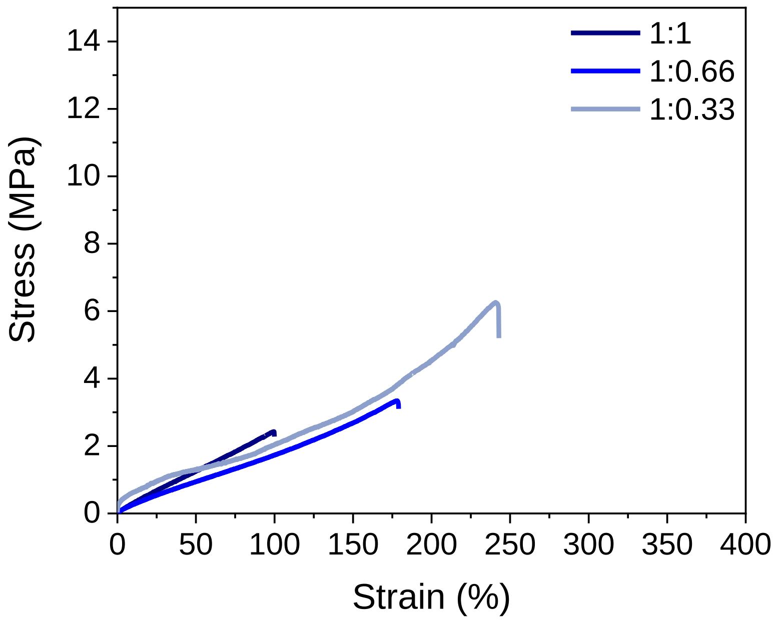

Fig. S16 Uniaxial tensile testing of perillyl itaconate (PerIt)/perillyl alcohol (PA):3T networks.

DSC data PerIt/PA:3T photosets - 1.5 wt.% of I819

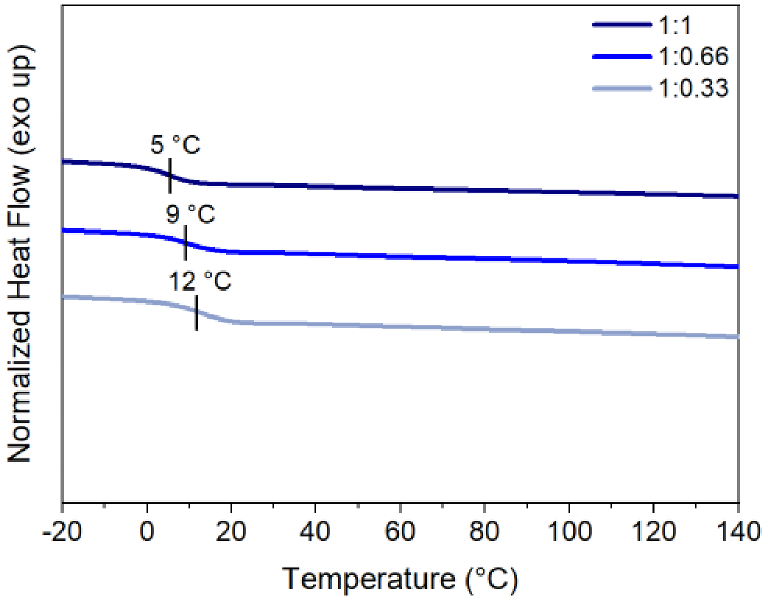

Fig. S17 DSC thermograms of PerIt/PA:3T networks at different thiol:ene ratios and 1.5 wt.% of I819. Second heating cycle from -20 to 140 °C.

DSC data PerIt/PA:3T photosets - 5 wt.% of I819

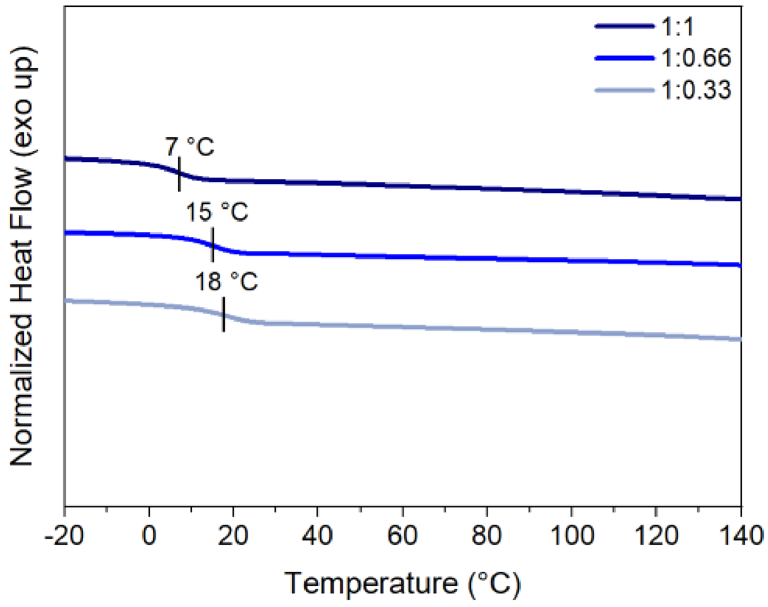

Fig. S18 DSC thermograms of PerIt/PA:3T networks at different thiol:ene ratios and 5 wt.% of I819. Second heating cycle from -20 to 140 °C.

TGA data PerIt/PA:3T photosets - 1.5 wt.% of I819

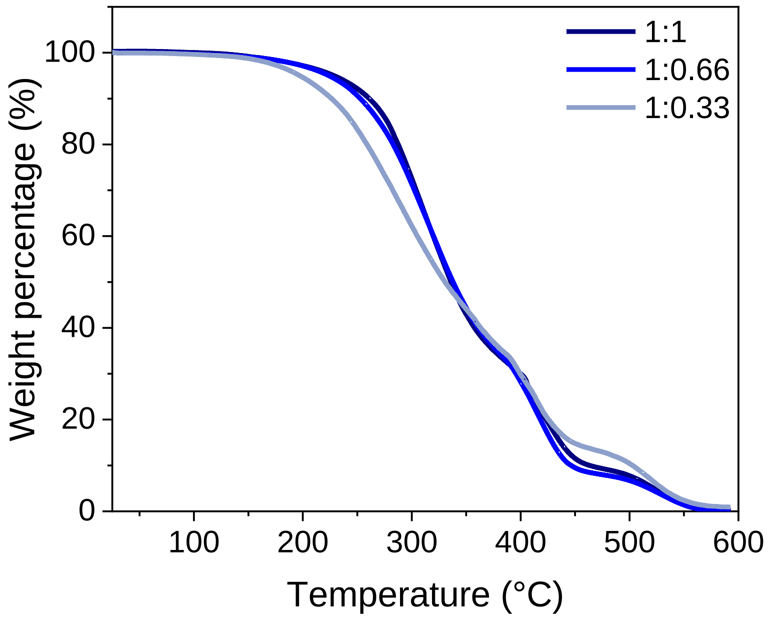

Fig. S19 TGA thermograms of PerIt/PA:3T networks at different thiol:ene ratios and 1.5 wt.% of I819.

TGA data PerIt/PA:3T photosets - 5 wt.% of I819

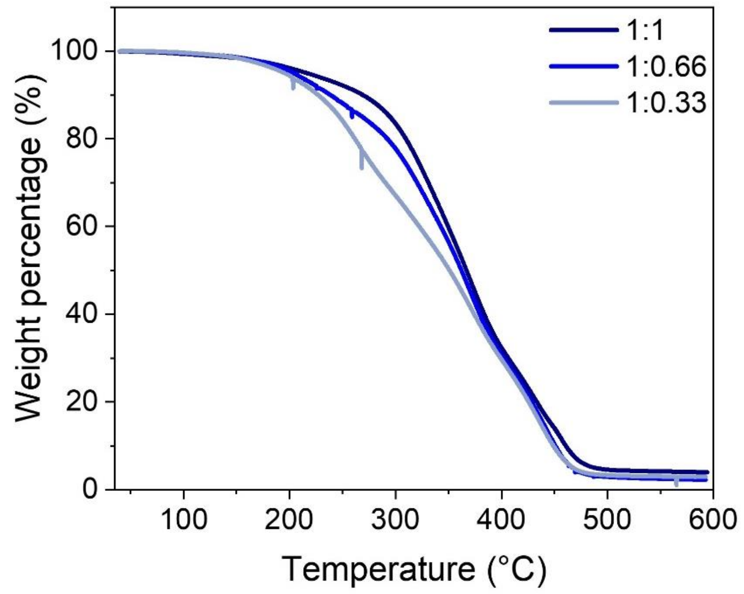

Fig. S20 TGA thermograms of PerIt/PA:3T networks at different thiol:ene ratios and 5 wt.% of I819.

DMA data PerIt/PA:3T photosets - 5 wt.% of I819

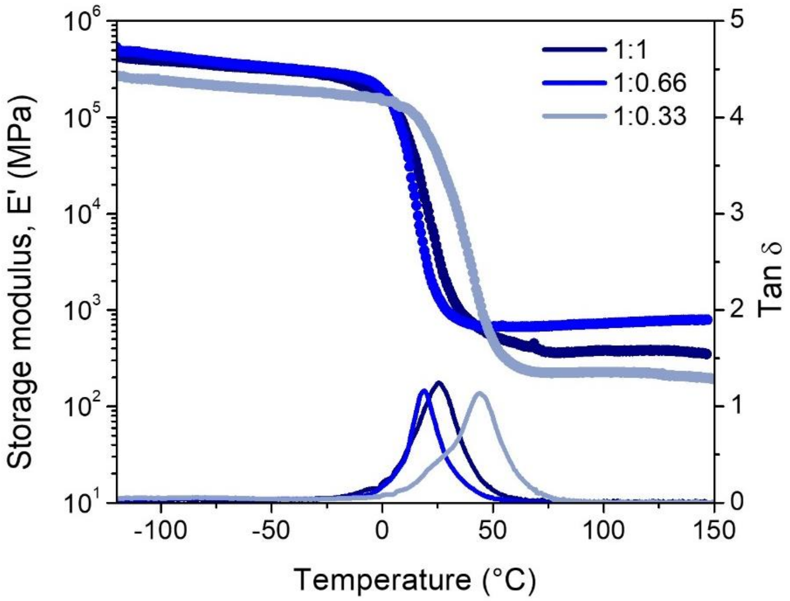

Fig. S21 DMA temperature sweep for PerIt/PA:3T networks at different thiol:ene ratios and 5 wt.% of I819.

PerIt/Lin monomer

<u>^{1}H NMR of PerIt/Lin monomer</u>

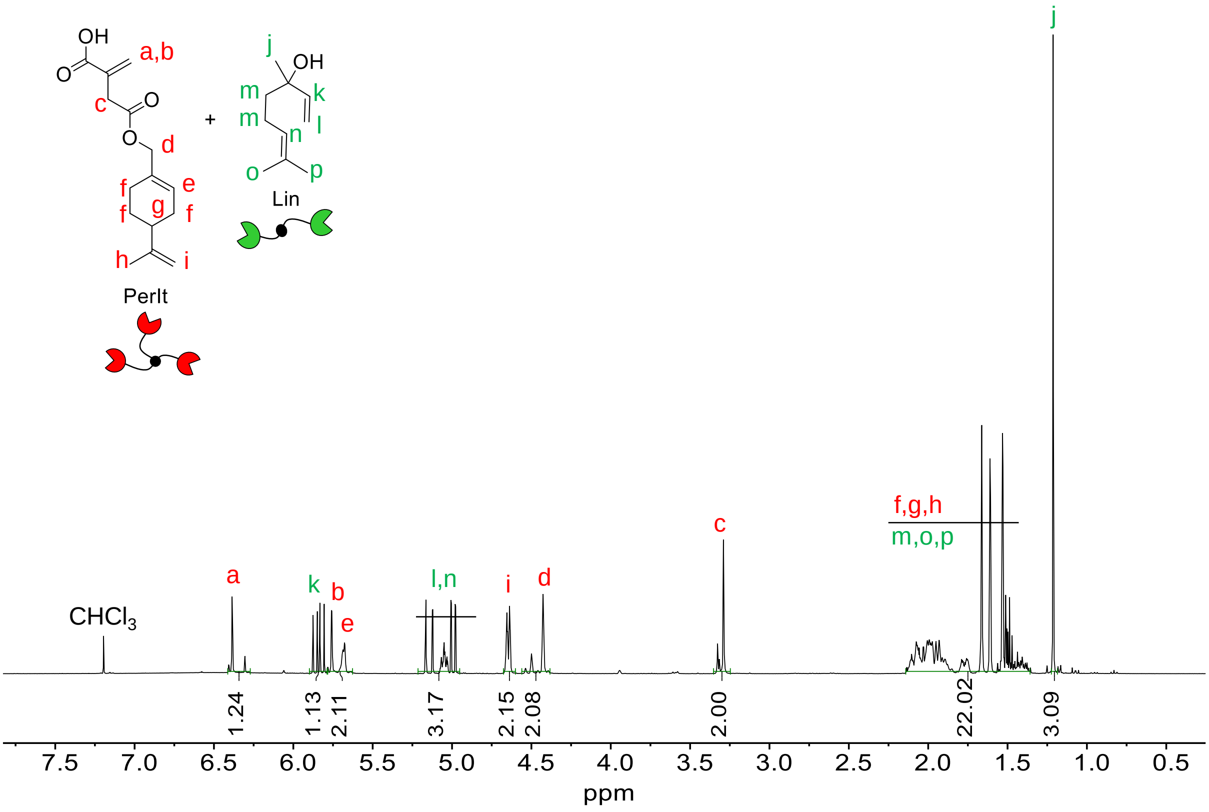

Fig. S22 ^{1}H NMR spectrum of perillyl itaconate/linalool monomer (300 MHz, 298 K, $CDCl_3$).

^{13}C NMR of PerIt/Lin monomer

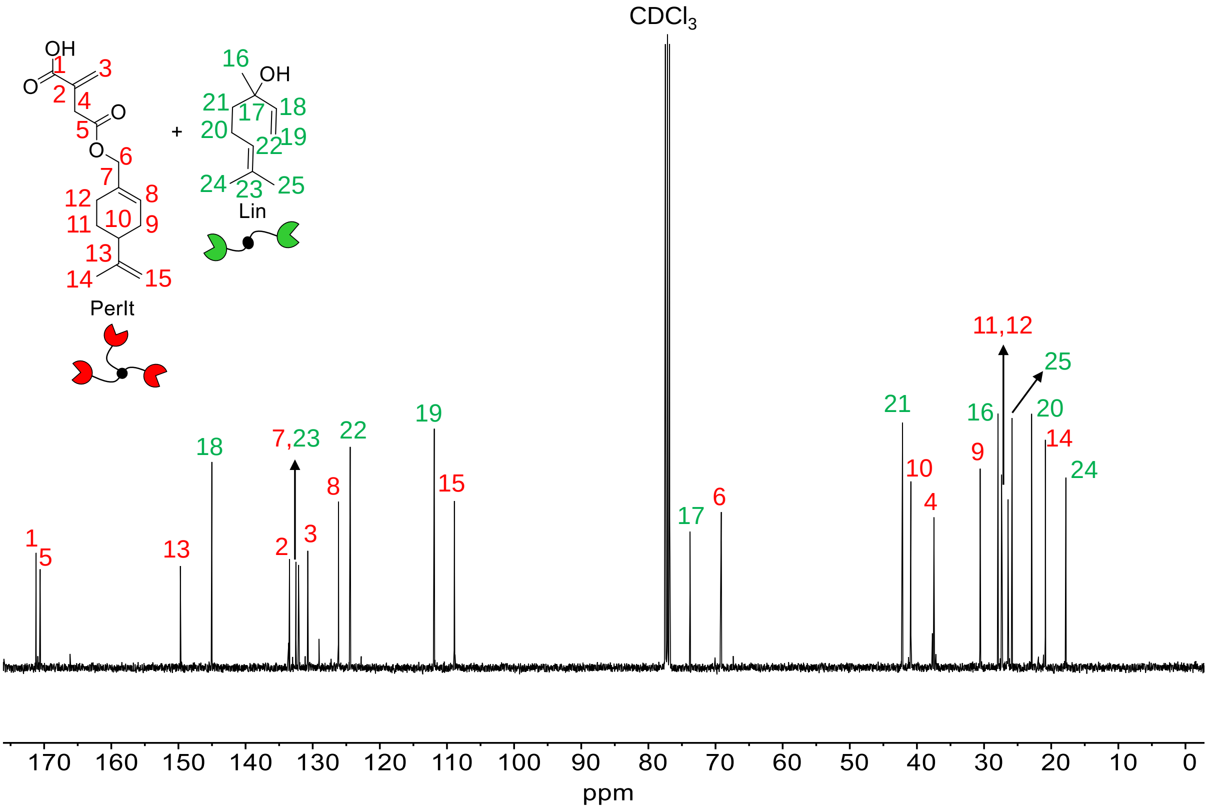

Fig. S23 ^{13}C NMR Spectrum of PerIt/Lin monomer - 101 MHz, 298 K, $CDCl_3$.

FT-IR spectra of PerIt/Lin monomer

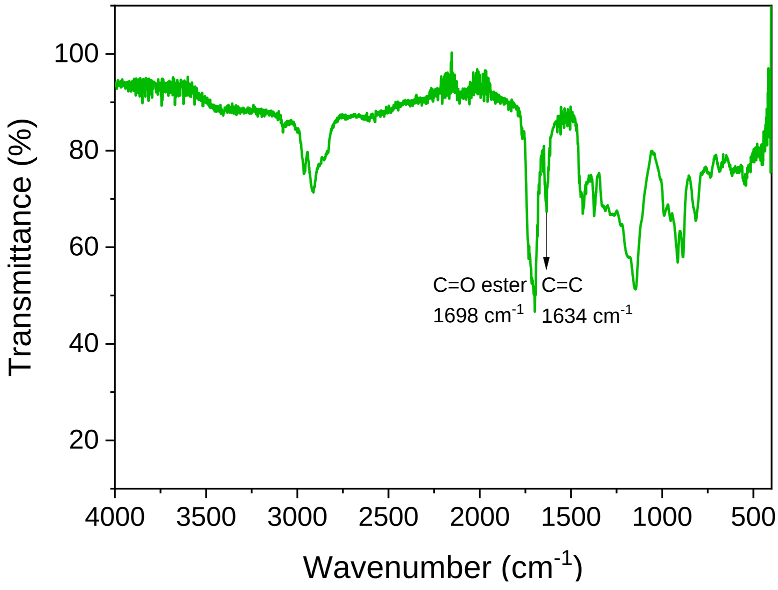

Fig. S24 FT-IR spectra of PerIt/Lin monomer.

FT-IR spectra of PerIt/Lin:3T photosets - 1.5 wt.% I819

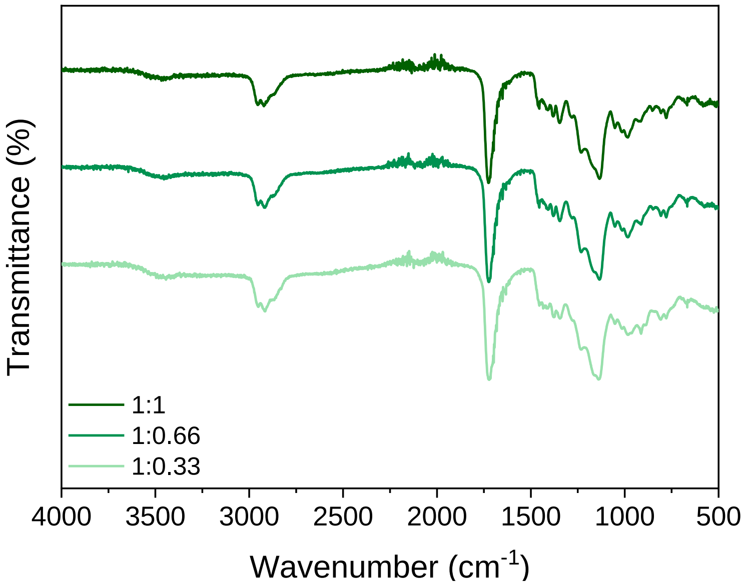

Fig. S25 FT-IR spectra of PerIt/Lin:3T networks (1.5 wt.% I819).

FT-IR spectra of PerIt/Lin:3T photosets - 5 wt.% I819

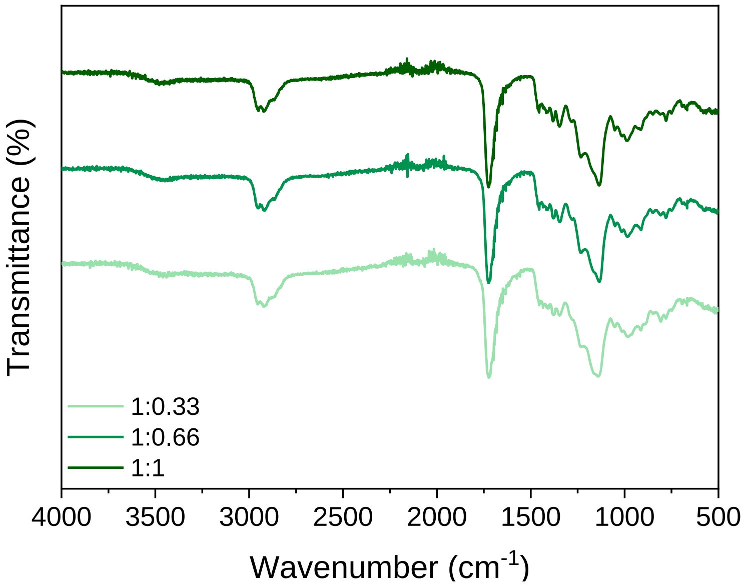

Fig. S26 FT-IR spectra of PerIt/Lin:3T networks (5 wt.% I819).

Tensile testing of PerIt/Lin:3T photosets - 1.5 wt.% of I819

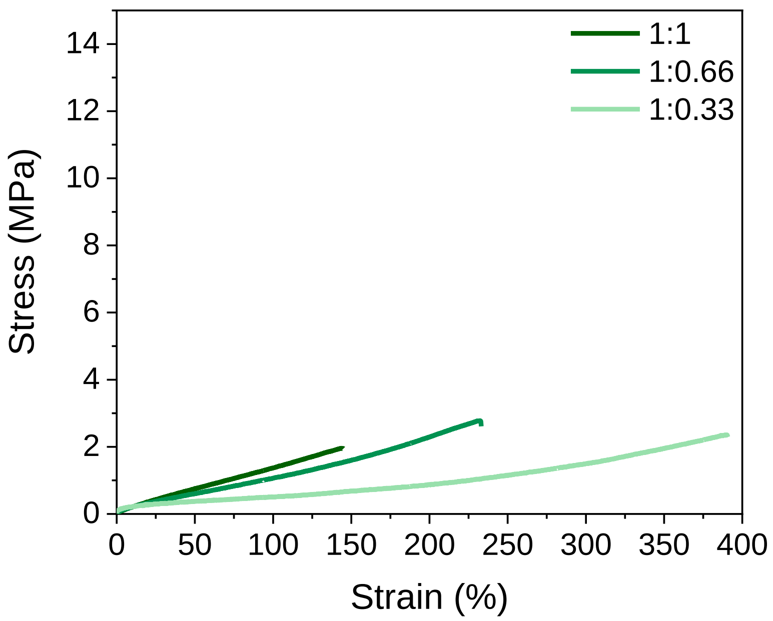

Fig. S27 Uniaxial tensile testing of perillyl itaconate (PerIt)/linalool (Lin):3T networks.

DSC data PerIt/Lin:3T photosets - 1.5 wt.% of I819

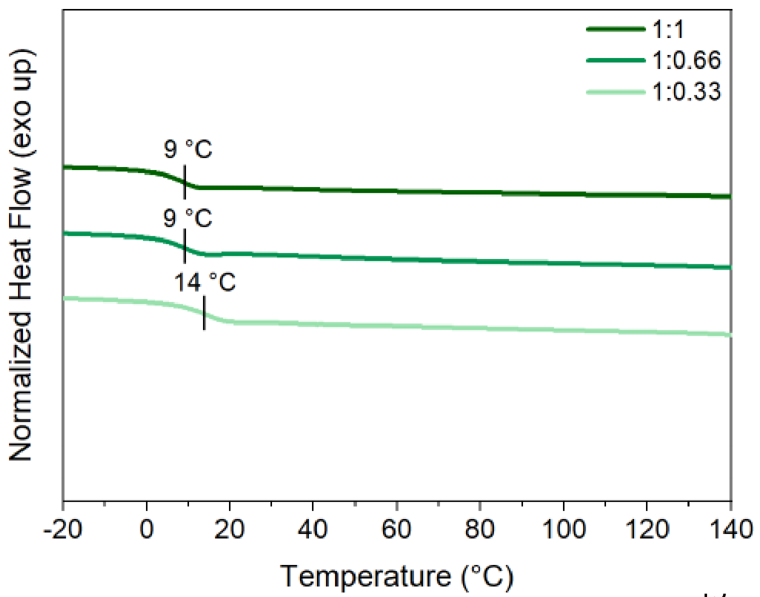

Fig. S28 DSC thermograms of PerIt/Lin:3T networks at different thiol:ene ratios and 1.5 wt.% of I819. Second heating cycle from -20 to 140 °C.

DSC data PerIt/Lin:3T photosets - 5 wt.% of I819

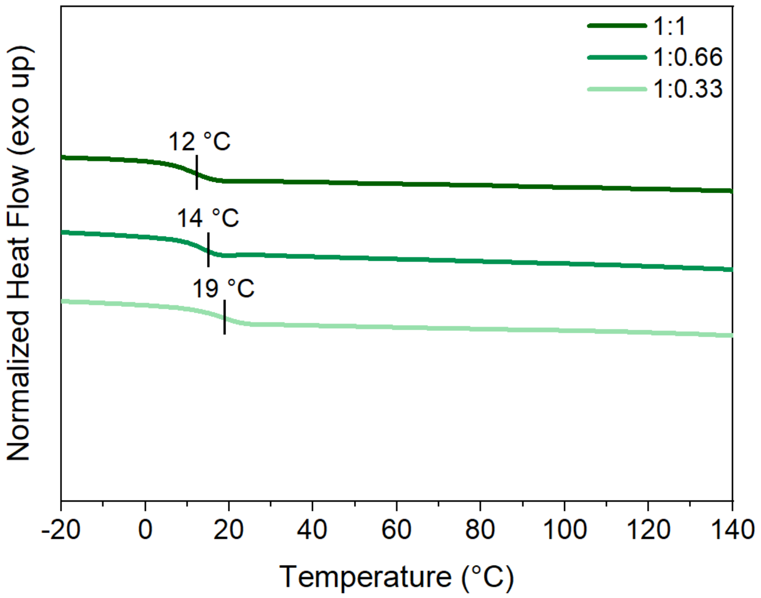

Fig. S29 DSC thermograms of PerIt/Lin:3T networks at different thiol:ene ratios and 5 wt.% of I819. Second heating cycle from -20 to 140 °C.

TGA data PerIt/Lin:3T photosets - 1.5 wt.% of I819

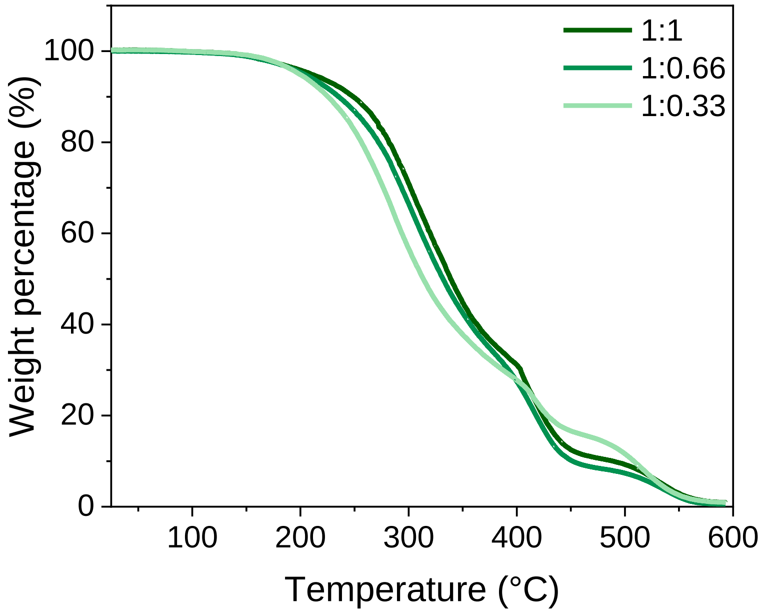

Fig. S30 TGA thermograms of PerIt/Lin:3T networks at different thiol:ene ratios and 1.5 wt.% of I819.

TGA data PerIt/Lin:3T photosets - 5 wt.% of I819

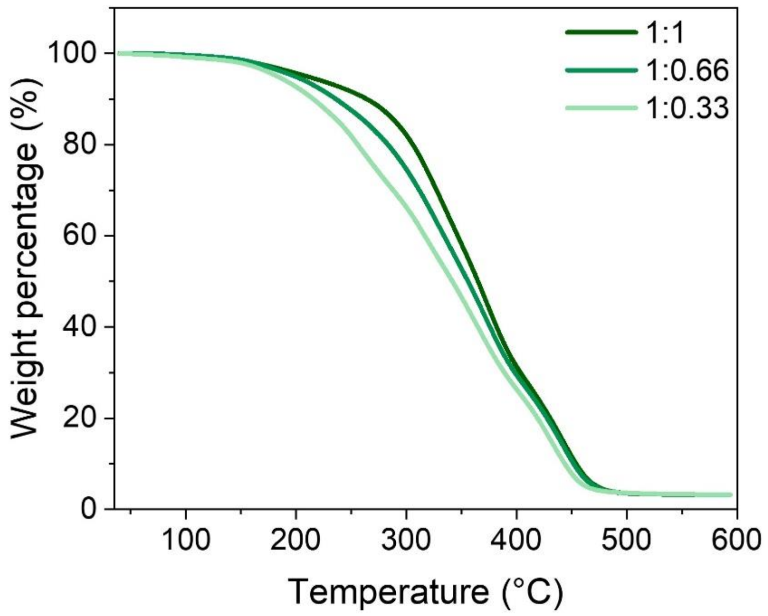

Fig. S31 TGA thermograms of PerIt/Lin:3T networks at different thiol:ene ratios and 5 wt.% of I819.

DMA data PerIt/Lin:3T photosets - 5 wt.% of I819

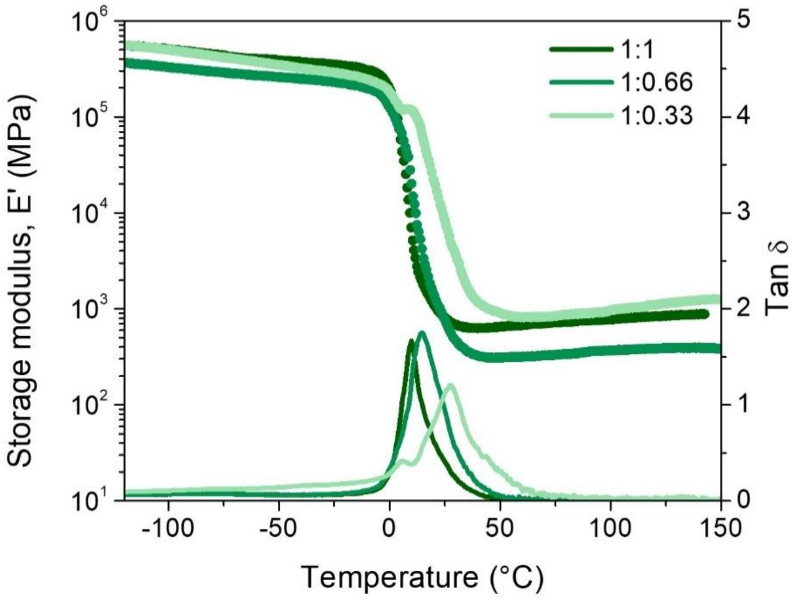

Fig. S32 DMA temperature sweep for PerIt/Lin:3T networks at different thiol:ene ratios and 5 wt.% of I819.

PerIt/Lim monomer

<u>^{1}H NMR of PerIt/Lim monomer</u>

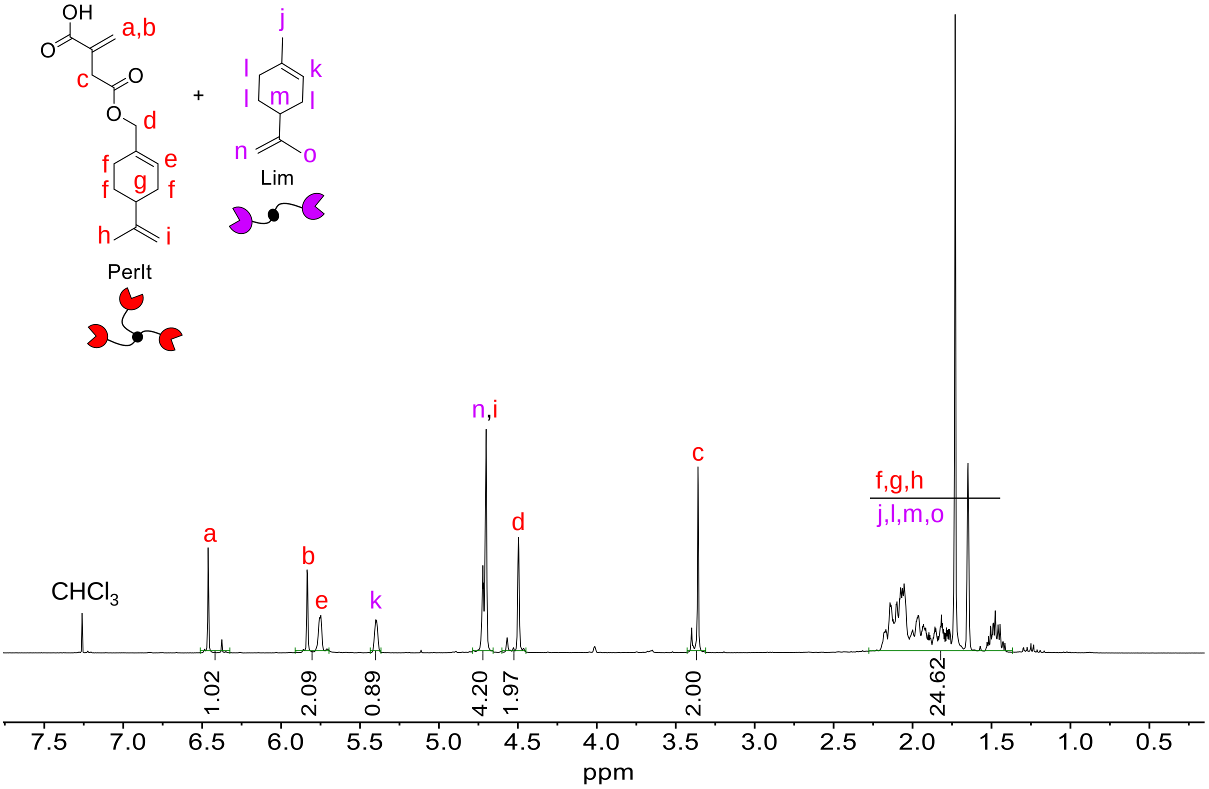

Fig. S33 ^{1}H NMR spectrum of perillyl itaconate/limonene monomer (300 MHz, 298 K, CDCl3).

^{13}C NMR of PerIt/Lim monomer

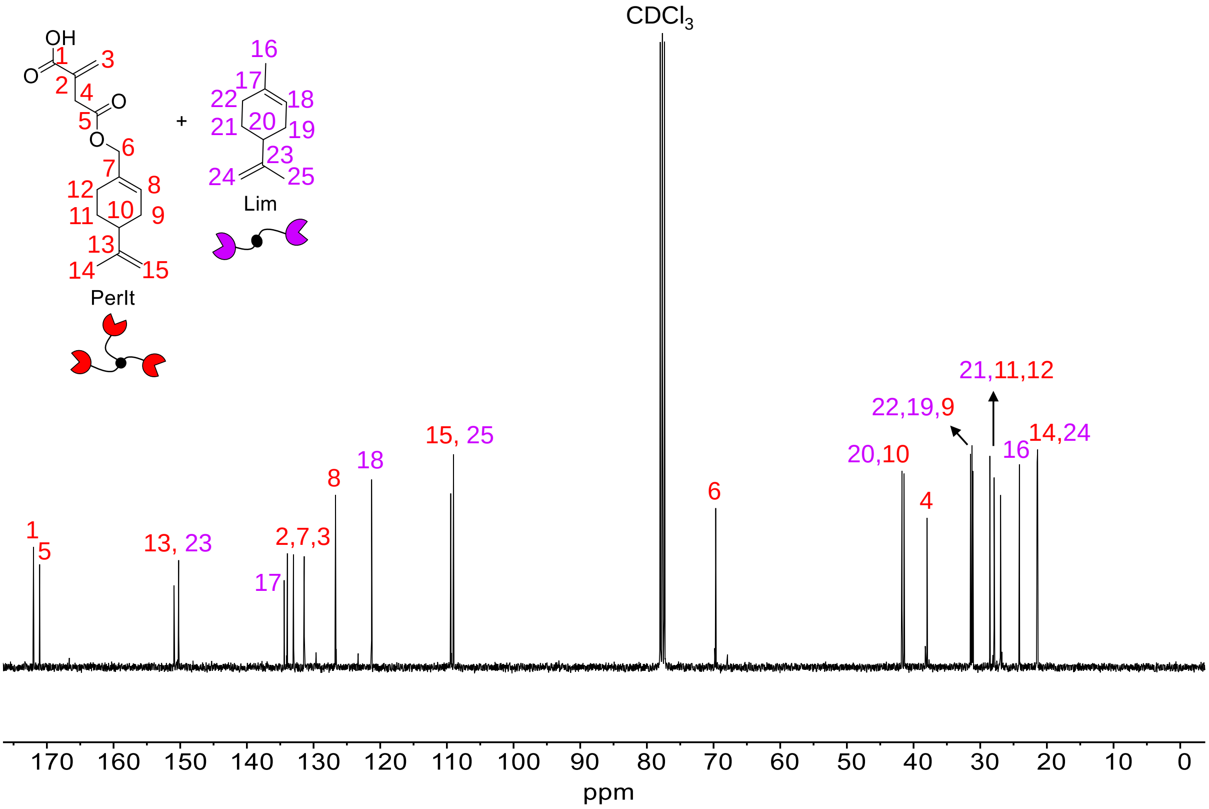

Fig. S34 ^{13}C NMR Spectrum of PerIt/Lim monomer - 101 MHz, 298 K, $CDCl_3$.

5 FT-IR spectra of PerIt/Lim monomer

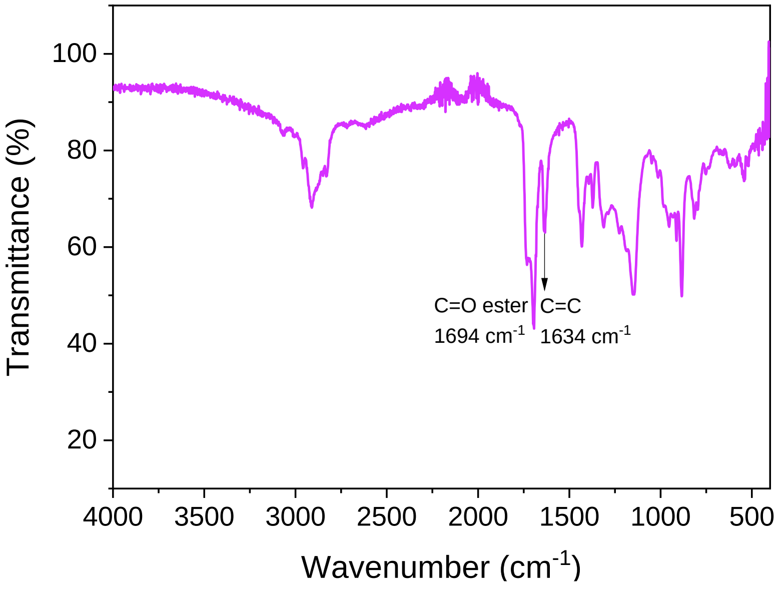

Fig. S35 FT-IR spectra of PerIt/Lim monomer.

FT-IR spectra of Perlt/Lim:3T photosets - 1.5 wt.% I819

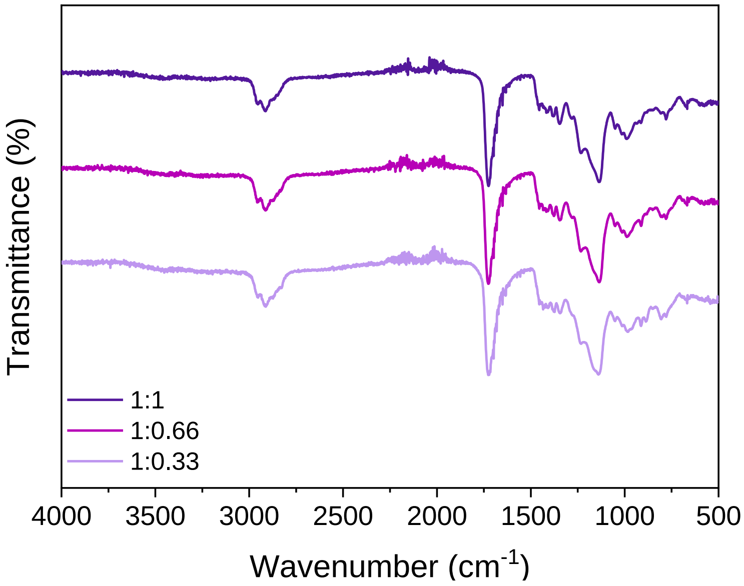

Fig. S36 FT-IR spectra of Perlt/Lim:3T networks (1.5 wt.% I819).

FT-IR spectra of Perlt/Lim:3T photosets - 5 wt.% I819

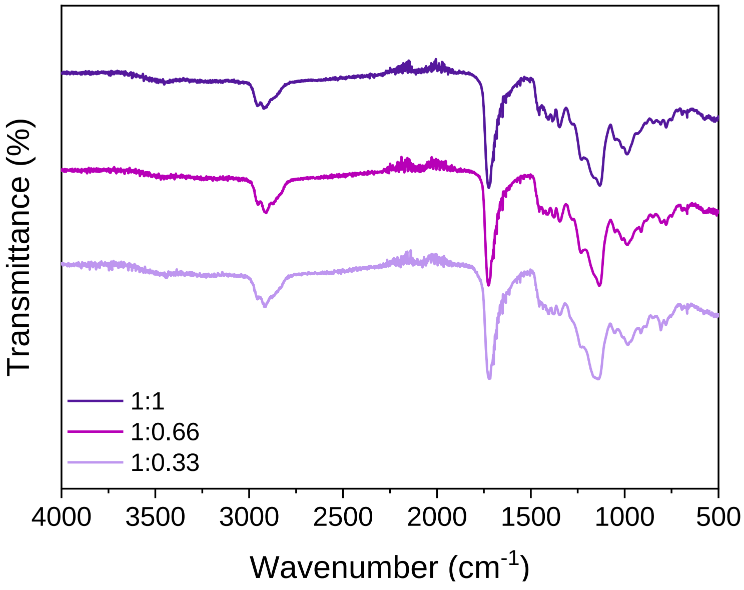

Fig. S37 FT-IR spectra of Perlt/Lim:3T networks (5 wt.% I819).

Tensile testing of PerIt/Lin:3T photosets - 1.5 wt.% of I819

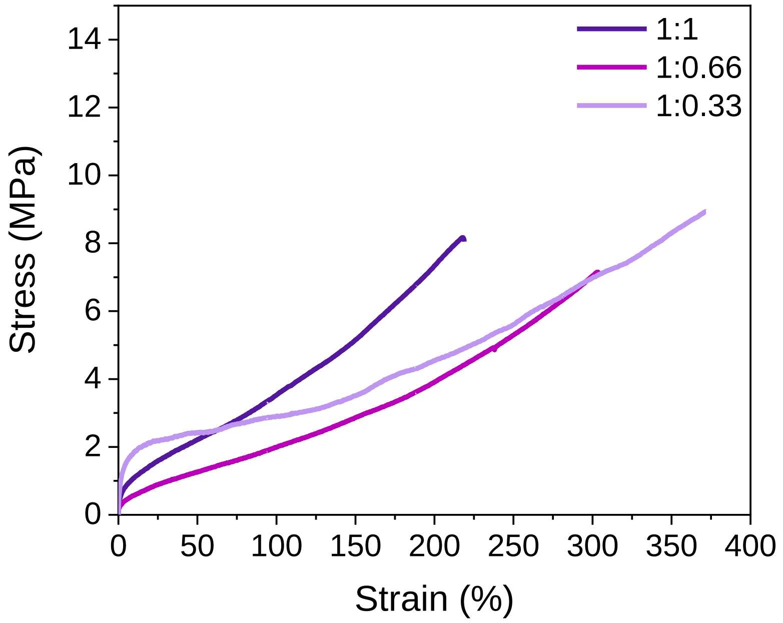

Fig. S38 Uniaxial tensile testing of perillyl itaconate (PerIt)/limonene (Lim):3T networks.

DSC data PerIt/Lim:3T photosets - 1.5 wt.% of I819

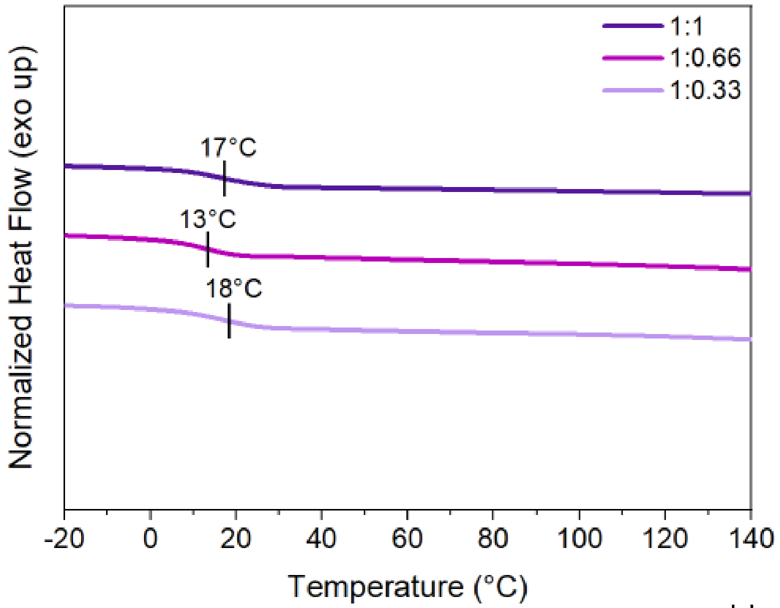

Fig. S39 DSC thermograms of PerIt/Lim:3T networks at different thiol:ene ratios and 1.5 wt.% of I819. Second heating cycle from -20 to 140 °C.

DSC data PerIt/Lim:3T photosets - 5 wt.% of I819

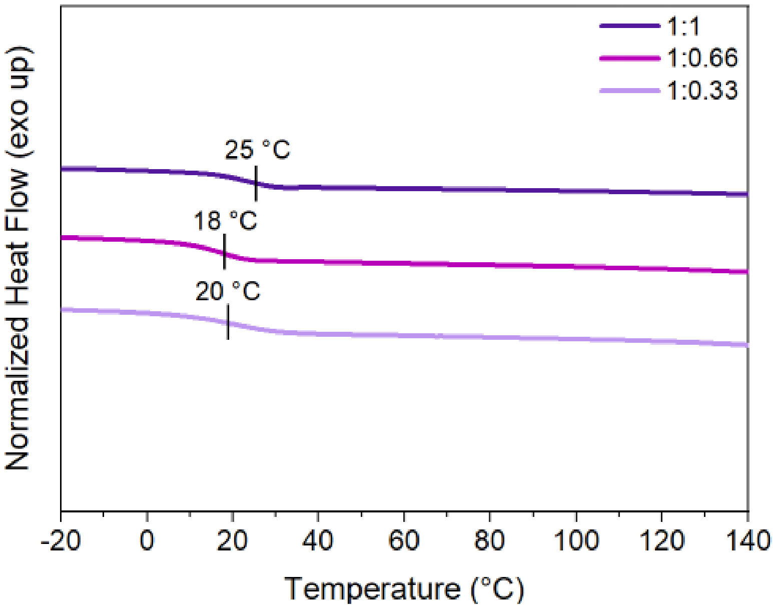

Fig. S40 DSC thermograms of PerIt/Lim:3T networks at different thiol:ene ratios and 5 wt.% of I819. Second heating cycle from -20 to 140 °C.

TGA data PerIt/Lim:3T photosets - 1.5 wt.% of I819

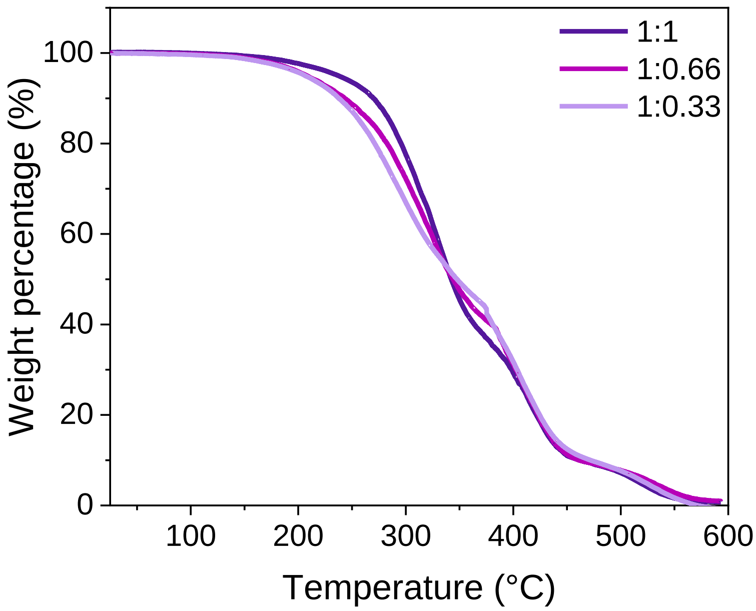

Fig. S41 TGA thermograms of PerIt/Lim:3T networks at different thiol:ene ratios and 1.5 wt.% of I819.

TGA data PerIt/Lim:3T photosets - 5 wt.% of I819

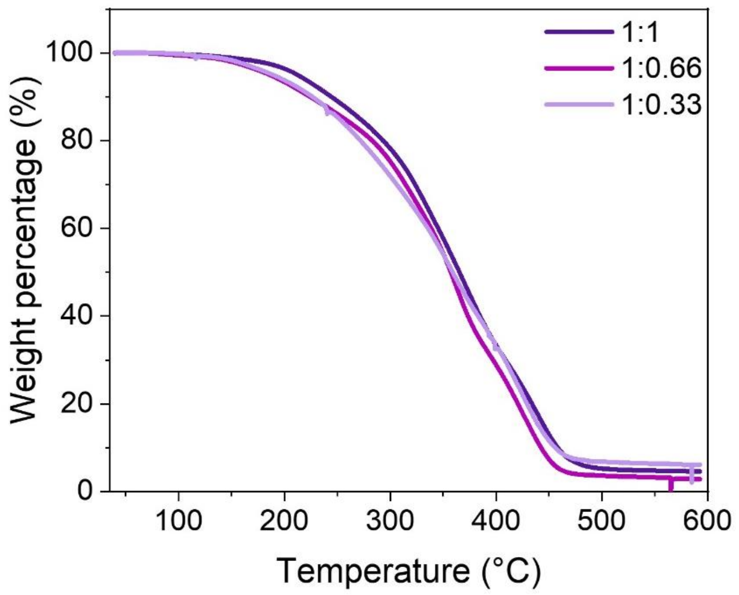

Fig. S42 TGA thermograms of PerIt/Lim:3T networks at different thiol:ene ratios and 5 wt.% of I819.

DMA data PerIt/Lim:3T photosets - 5 wt.% of I819

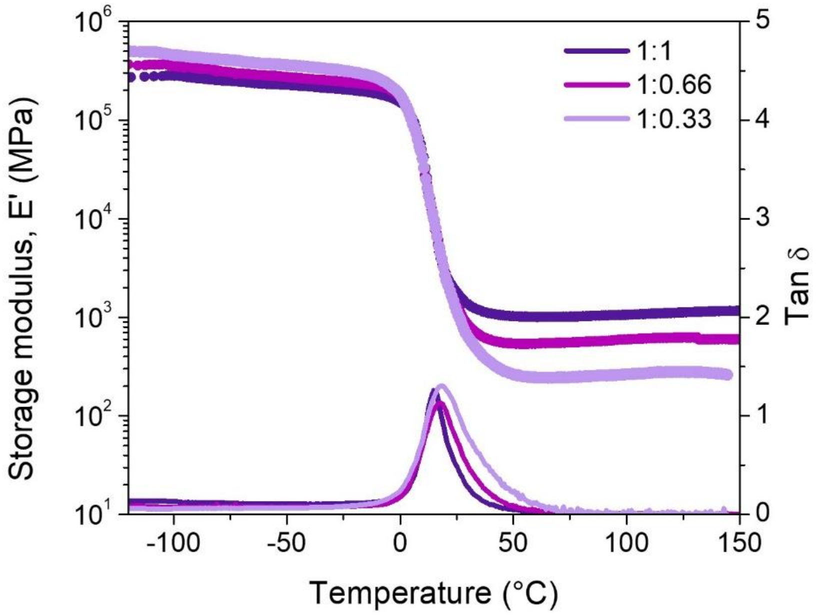

Fig. S43 DMA temperature sweep for PerIt/Lim:3T networks at different thiol:ene ratios and 5 wt.% of I819.

Photoreology of PerIt/Lim:3T using 1.5 wt.% of I819

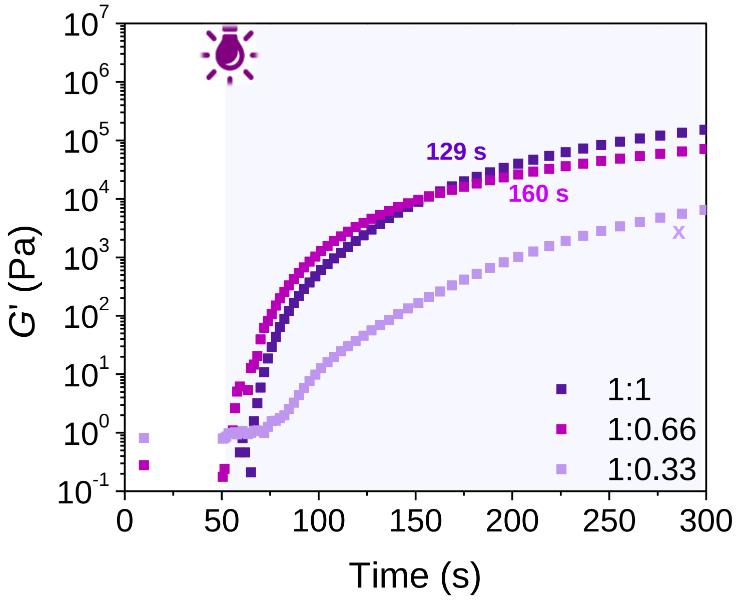

Fig. S44 Photorheology of PerIt/Lim:3T at different thiol:ene ratios using 1.5 wt.% of photoinitiator under oscillatory shear at room temperature.

Photoreology of PerIt/Lim:3T using 5 wt.% of I819

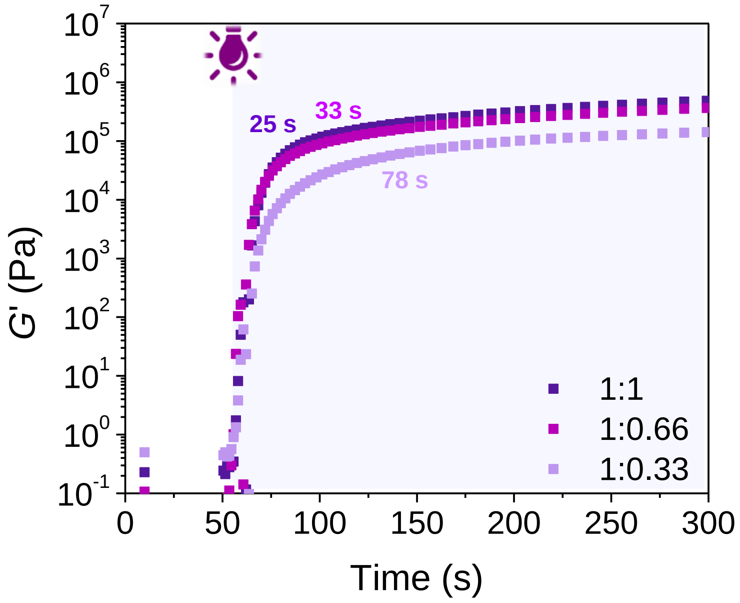

Fig. S45 Photorheology of PerIt/Lim:3T at different thiol:ene ratios using 5 wt.% of photoinitiator under oscillatory shear at room temperature.

PerIt/Lim:3T resins

<u>3D printing: resin containing BHT</u>

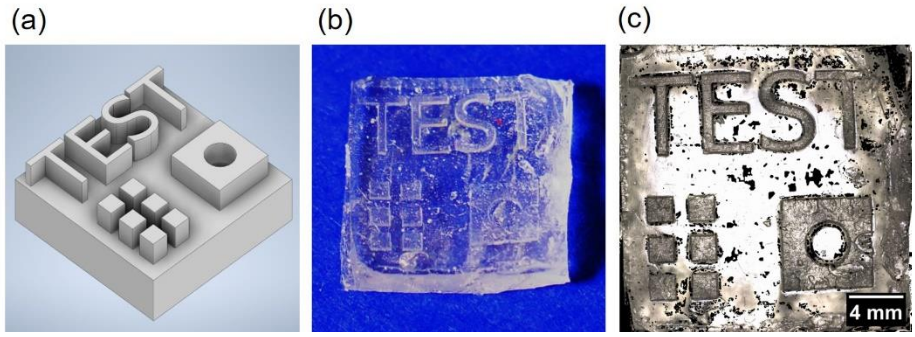

Fig. S46 (a) 3D CAD model of a square test used for precise DLP. (b) Photograph of 3D-printed part from PerIt/Lim-3T 1:1, 5 wt.% of I819 and 1 wt.% of BHT. Printing conditions: 50 μm and 90 s cure/layer. (C) Microscope image of the printed part.

<u>Viscosity of PerIt/Lim:3T resins</u>

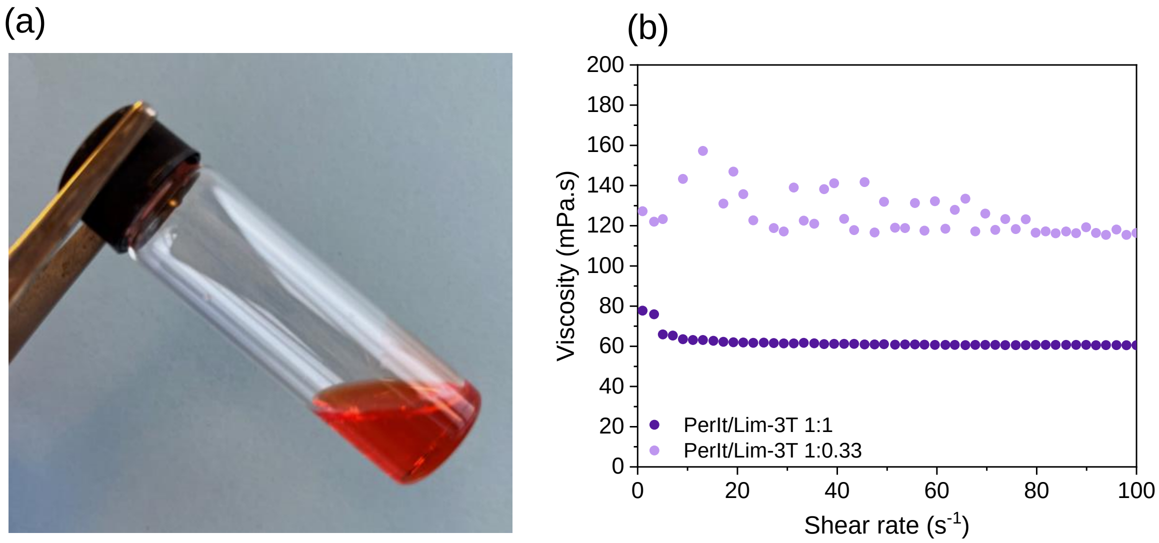

Fig. S47 PerIt/Lim:3T resins containing additives. (a) 5 wt.% of I819, 1 wt.% of BHT and 0.03 wt.% of Sudan Red II. (b) Resin viscosity between a shear rate of 1 to 100 s-1 for PerIt/Lim:3T 1:1 (5 wt.% of I819, 1 wt.% of BHT and 0.03 wt.% of Sudan Red II) and PerIt/Lim:3T 1:0.33 (7 wt.% of I819, 2 wt.% BHT and 0.06 wt.% of Sudan Red II).

Z-axis cure depth

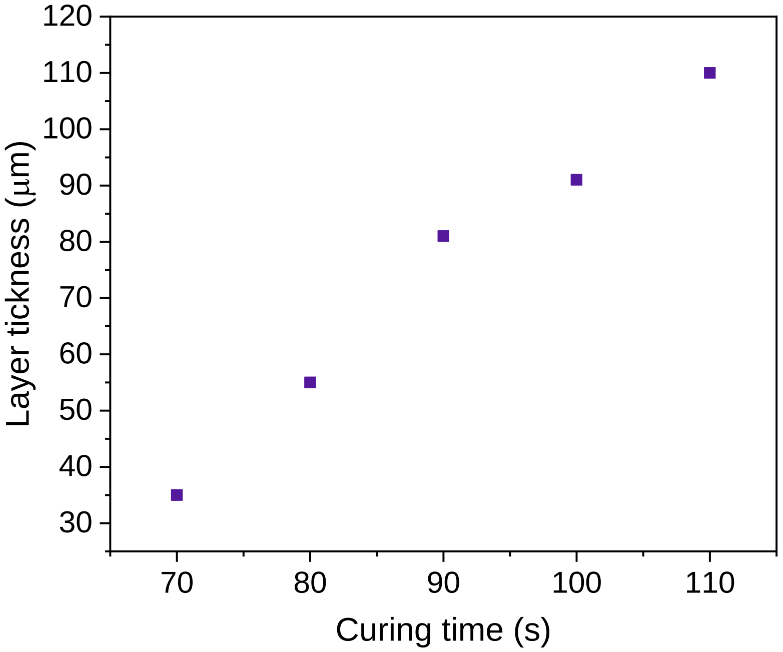

Fig. S48 Cure depth versus exposure time for PerIt/Lim:3T 1:1 (5 wt.% of I819, 1 wt.% of BHT and 0.03 wt.% of Sudan Red II).

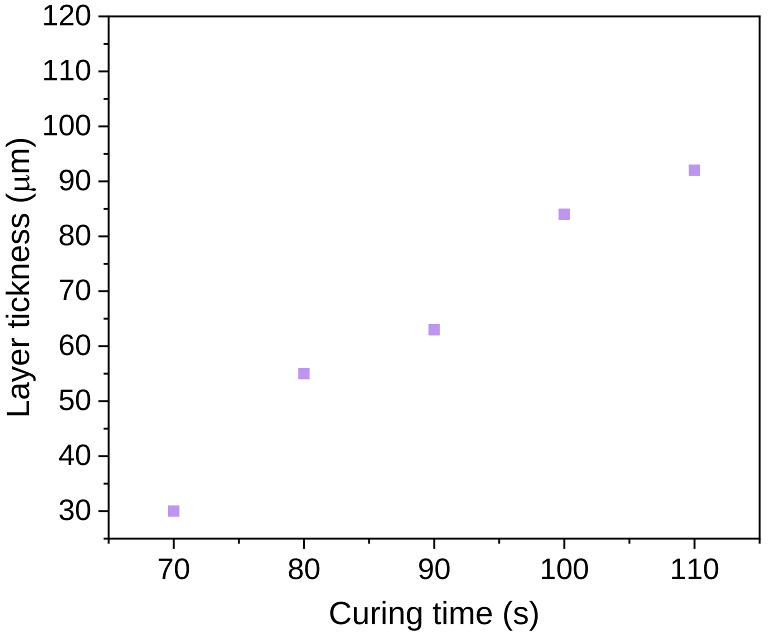

Fig. S49 Cure depth versus exposure time for PerIt/Lim:3T 1:0.33 (7 wt.% of I819, 2 wt.% BHT and 0.06 wt.% of Sudan Red II).

3D printing: resins containing BHT and Sudan Red II

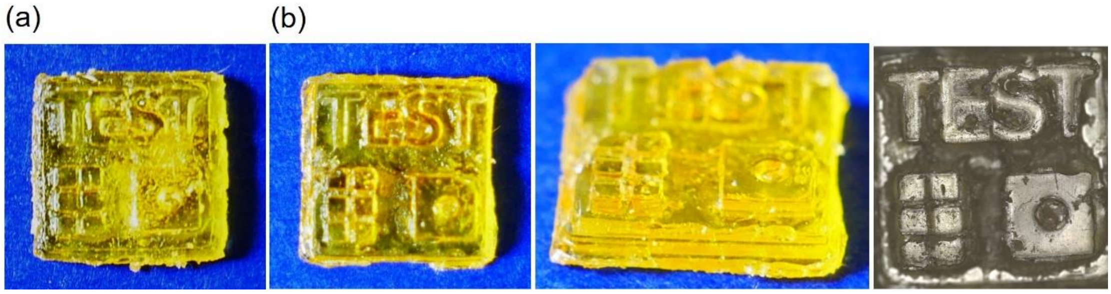

Fig. S50 (a) 3D-printed part from a resin containing PerIt/Lim-3T in 1:1 ratio, 5 wt.% of I819, 1 wt.% of BHT and 0.01 wt.% of Sudan Red II. Printing conditions: (A) 25 µm and 100 s cure/layer and (B) 50 µm and 110 s cure/layer.

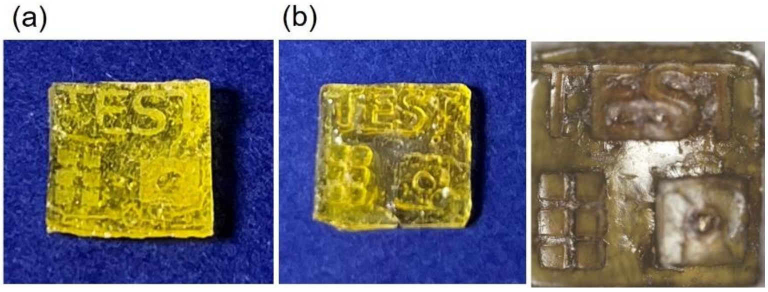

Fig. S51 (a) 3D-printed part from a resin containing PerIt/Lim-3T in 1:0.33 ratio, 7 wt.% of I819, 2 wt.% of BHT and 0.06 wt.% of Sudan Red II. Printing conditions: (A) 25 µm and 100 s cure/layer and (B) 50 µm and 110 s cure/layer.

Table S1. Comparative of mechanical properties of resins formulated with purified and non-purified PerIt. Formulation containing PerIt:3T 1:1 and 1.5 wt.% of I819.

Network	UTS (MPa)	ε_{break} (%)	*E* (MPa)
Crude PerIt	11.9 ± 0.4	106 ± 10	135.5 ± 13.5
Purified PerIt	9.6 ± 1.5	111 ± 12	109.0 ± 27.5

Table S2. Summary of thermomechanical data and gel fraction for all designed networks.

Network	PerIt or PerIt/dil (wt.%)	T_g (°C)	$T_{d,5\%}$ (°C)	UTS (MPa)	ε_{break} (%)	E (MPa)	Gel fraction (%)
1.5 wt.% I819							
PerIt:3T 1:1	39.3	19	226	11.9 ± 0.4	106 ± 10	135.5 ± 13.5	100 ± 4.2
PerIt:3T 1:0.66	49.1	24	206	12.3 ± 2.3	97 ± 30	196.7 ± 20.2	100 ± 3.8
PerIt:3T 1:0.33	65.6	36	191	28.0 ± 1.5	30 ± 2	358.0 ±15.1	91 ± 8.0
PerIt/PA:3T 1:1	37.5	5	228	2.3 ± 0.1	98 ± 1	3.6 ± 0.5	100 ± 2.2
PerIt/PA:3T 1:0.66	47.3	9	223	2.8 ± 0.4	140 ± 27	3.8 ± 0.3	98 ± 1.6
PerIt/PA:3T 1:0.33	64.0	12	197	4.6 ± 1.2	254 ± 12	14.6 ± 6.5	95 ± 1.0
PerIt/Lin:3T 1:1	39.6	9	209	1.8 ± 0.1	139 ± 9	2.5 ± 0.7	100 ± 3.2
PerIt/Lin:3T 1:0.66	49.8	9	203	2.6 ± 0.1	236 ± 3	3.7 ± 0.2	74 ± 1.3
PerIt/Lin:3T 1:0.33	67.5	14	198	2.2 ± 0.2	367 ± 23	7.7 ± 0.8	72 ± 8.1
PerIt/Lim:3T 1:1	37.8	17	236	7.5 ± 0.5	216 ± 2	39.1 ± 0.9	100 ± 4.5
PerIt/Lim:3T 1:0.66	48.0	13	208	6.6 ± 0.5	291 ± 9	21.3 ± 1.9	96 ± 6.3
PerIt/Lim:3T 1:0.33	65.8	18	206	8.7 ± 0.2	344 ± 19	78.4 ± 1.3	84 ± 1.0
5 wt.% I819							
PerIt:3T 1:1	38.0	19	219	11.6 ± 2.3	151 ± 2	61.5 ± 10.3	100 ± 1.3
PerIt:3T 1:0.66	47.5	33	195	18.8 ± 3.2	97 ± 6	270 ± 58.0	100 ± 2.1
PerIt:3T 1:0.33	63.4	43	186	30.5 ± 2.6	15 ± 1	247.5 ± 97.5	72 ± 7.9
PerIt/PA:3T 1:1	36.2	7	216	2.7 ± 0.5	101 ± 4	5.0 ± 0.9	91 ± 0.7
PerIt/PA:3T 1:0.66	45.7	15	203	5.3 ± 0.3	167 ± 7	10.4 ± 1.2	98 ± 6.6
PerIt/PA:3T 1:0.33	61.9	18	195	7.7 ± 0.7	244 ± 2	41.4 ± 0.7	94 ± 3.2
PerIt/Lin:3T 1:1	38.2	12	209	3.3 ± 0.3	158 ± 3	6.3 ± 1.0	81 ± 6.4
PerIt/Lin:3T 1:0.66	48.2	14	199	3.1 ± 0.2	203 ± 12	7.7 ± 0.4	100 ± 6.4
PerIt/Lin:3T 1:0.33	65.2	19	183	4.1 ± 0.1	353 ± 2	21.5 ± 3.5	78 ± 6.5
PerIt/Lim:3T 1:1	36.6	25	212	10.6 ± 0.4	220 ± 3	56.9 ± 4.5	100 ± 11.6
PerIt/Lim:3T 1:0.66	46.4	18	186	6.3 ± 0.8	321 ± 33	15.9 ± 2.7	98 ± 1.2
PerIt/Lim:3T 1:0.33	63.6	20	189	7.2 ± 1.8	242 ± 10	69.1 ± 17.5	87 ± 1.4

Table S3. Mechanical properties for commercial 3D printing resins.

Supplier	Resin code	UTS (MPa)	E (MPa)	ε_{break} (%)	Standard
Photocentric[1]	Flexible UV160TR	14.5	66.5	160	ASTM D412
FormLabs[2]	Elastic 50A	3.23	-	160	ASTM D 412-6 (A)
FormLabs[3]	Flexible 80A	8.9	-	120	ASTM D 412-06 (A)

Table S4. Summary of DMA data for networks designed with 5 wt.% of I819.

Network	E'_{glassy} (MPa)[a]	$E'_{rubbery}$ (MPa)[b]	Tan delta peak (°C)	M_c (g/mol)[c]	q^d
PerIt:3T 1:1	311300	1002	27	10.0	34.4
PerIt:3T 1:0.66	530340	2322	30	4.3	76.5
PerIt:3T 1:0.33	330110	630	44	15.9	19.2
PerIt/PA:3T 1:1	316140	378	25	26.6	12.3
PerIt/PA:3T 1:0.66	330860	780	18	12.9	23.9
PerIt/PA:3T 1:0.33	194650	210	44	47.9	6.1
PerIt/Lin:3T 1:1	388950	856	10	11.7	27.6
PerIt/Lin:3T 1:0.66	259290	392	14	25.7	11.7
PerIt/Lin:3T 1:0.33	346820	1163	27	8.6	31.2
PerIt/Lim:3T 1:1	226890	1135	14	8.9	36.5
PerIt/Lim:3T 1:0.66	274050	625	17	16.1	18.8
PerIt/Lim:3T 1:0.33	362000	279	18	36.1	7.6

[a] Glassy modulus obtained at - 50 °C. [b] Rubbery modulus obtained at 130 °C.

Molecular weight between crosslinks (M_c)[c] and crosslinking density (q)[d] were calculated following the classical theory of rubber elasticity.[4,5] $E'_{rubbery}$ can be correlated with M_c according to equation 1:

$$M_c = \frac{3RTd}{E'_{rubbery}} \qquad (1)$$

where T is the absolute temperature at which the modulus is determined, d is polymer density (d = 1 g/mL was used for all obtained networks), and R is the universal gas constant. From this, M_c can be transferred into the crosslink density (q):

$$M_c = \frac{MW}{q} \qquad (2)$$

where MW is the monomer molecular weight. For each network formulation, an average molecular weight was calculated based on the concentration of each resin component (monomers and thiol).

References

[1] Photocentric Group. https://photocentricgroup.com/wp-content/uploads/2023/01/TDS-Flexible-UV160TR-Resin-RV1.pdf (accessed 2024-03-19).

[2] formlabs. https://formlabs-media.formlabs.com/datasheets/2001420-TDS-ENUS-0.pdf (accessed 2024-03-19).

[3] formlabs. https://formlabs-media.formlabs.com/datasheets/2001418-TDS-ENUS-0.pdf (accessed 2024-03-19).

[4] TA instruments. www.tainstruments.com/pdf/literature/RH102.pdf (accessed 2024-03-19)

[5] Barszczewska-Rybarek, I. M.; Korytkowska-Wałach, A.; Kurcok, M.; Chladek, G.; Kasperski, J., DMA analysis of the structure of crosslinked poly(methyl methacrylate)s. *Acta Bioeng. Biomech.* **2017**, 19, 47-53.
